# Enantiospecific Synthesis, Chiral Separation, and Biological Activity of Four Indazole-3-Carboxamide-Type Synthetic Cannabinoid Receptor Agonists and Their Detection in Seized Drug Samples

**DOI:** 10.3389/fchem.2019.00321

**Published:** 2019-05-16

**Authors:** Lysbeth H. Antonides, Annelies Cannaert, Caitlyn Norman, Loelia Vives, Aidan Harrison, Andrew Costello, Niamh Nic Daeid, Christophe P. Stove, Oliver B. Sutcliffe, Craig McKenzie

**Affiliations:** ^1^Leverhulme Research Centre for Forensic Science, University of Dundee, Dundee, United Kingdom; ^2^Forensic Drug Research Group, Centre for Anatomy and Human Identification, University of Dundee, Dundee, United Kingdom; ^3^Laboratory of Toxicology, Faculty of Pharmaceutical Sciences, Ghent University, Ghent, Belgium; ^4^Laboratory of Toxicology, National Institute of Criminalistics and Criminology, Brussels, Belgium; ^5^IUT “A” Paul Sabatier, Département de Chimie, Castres, France; ^6^Phenomenex, Macclesfield, United Kingdom; ^7^Manchester Drug Analysis and Knowledge Exchange, Manchester Metropolitan University, Manchester, United Kingdom; ^8^Greater Manchester Police, Manchester, United Kingdom; ^9^Division of Chemistry and Environmental Science, Manchester Metropolitan University, Manchester, United Kingdom

**Keywords:** synthetic cannabinoid receptor agonists, chiral, pharmacology, bioassay, detection

## Abstract

Synthetic cannabinoid receptor agonists (SCRAs) have been the largest group of illicit psychoactive substances reported to international monitoring and early warning systems for many years. Carboxamide-type SCRAs are amongst the most prevalent and potent. Enantiospecific synthesis and characterization of four indazole-3-carboxamides, AMB-FUBINACA, AB-FUBINACA, 5F-MDMB-PINACA (5F-ADB), and AB-CHMINACA is reported. The interactions of the compounds with CB_1_ and CB_2_ receptors were investigated using a G-protein coupled receptor (GPCR) activation assay based on functional complementation of a split NanoLuc luciferase and EC_50_ (a measure of potency) and E_max_ (a measure of efficacy) values determined. All compounds demonstrated higher potency at the CB_2_ receptor than at the CB_1_ receptor and (*S*)-enantiomers had an enhanced potency to both receptors over the (*R*)-enantiomers. The relative potency of the enantiomers to the CB_2_ receptor is affected by structural features. The difference was more pronounced for compounds with an amine moiety (AB-FUBINACA and AB-CHMINACA) than those with an ester moiety (AMB-FUBINACA and 5F-MDMB-PINACA). An HPLC method was developed to determine the prevalence of (*R*)-enantiomers in seized samples. Lux® Amylose-1 [Amylose tris(3,5-dimethylphenylcarbamate)] has the greatest selectivity for the SCRAs with a terminal methyl ester moiety and a Lux® i-Cellulose-5 column for SCRAs with a terminal amide moiety. Optimized isocratic separation methods yielded enantiomer resolution values (Rs) ≥ 1.99. Achiral GC-MS analysis of seized herbal materials (*n* = 16), found 5F-MDMB-PINACA (<1.0–91.5 mg/g herbal material) and AMB-FUBINACA (15.5–58.5 mg/g herbal material), respectively. EMB-FUBINACA, AMB-CHMICA, 5F-ADB-PINACA isomer 2, and ADB-CHMINACA were also tentatively identified. Analysis using chiral chromatography coupled to photodiode array and quadrupole time of flight mass spectrometry (chiral HPLC-PDA-QToF-MS/MS) confirmed that the (*S*)-enantiomer predominated in all samples (93.6–99.3% (S)-enantiomer). Small but significant differences in synthesis precursor enantiopurity may provide significant differences between synthesis batches or suppliers and warrants further study. A method to compare potency between samples containing different SCRAs at varying concentrations was developed and applied in this small preliminary study. A 10-fold difference in the “intrinsic” potency of samples in the study was noted. With the known heterogeneity of SCRA infused materials, the approach provides a simplified method for assessing and communicating the risk of their use.

## Introduction

Synthetic cannabinoids (SCs) are the most chemically diverse group of new psychoactive substances (NPS) reported to national and international drug early warning systems (EMCDDA, 2018a; UNODC, 2018a). Synthetic cannabinoid receptor agonists (SCRAs) are cannabimimetics, exerting their pharmacological effects through human cannabinoid type 1 and type 2 (CB_1_ and CB_2_) G-Protein Coupled Receptors (GPCRs). Due to their structural diversity they exhibit a wide range of receptor binding and activation properties and therefore have a wide range of potency and efficacy. CB_1_ receptors, present primarily in the central nervous system and to a lesser extent in the peripheral tissues, mediate the psychoactive effects associated with SCRAs whilst CB_2_ receptors are expressed primarily in the immune system (Pertwee, 1997, 2008; Pertwee et al., 2010). CB_1_ and CB_2_ receptor agonists are recognized for their potential as therapeutic agents (Han et al., 2013). As a result, many SCRA classes and hundreds of analogs have been synthesized and studied as pharmacological targets by research groups across academia and industry. These compounds have often been found to bind to and activate both receptors and, as a result of their psychoactive effects, few have reached clinical trials and have been developed as medicinal products (e.g., Huffman and Padgett, 2005; Huffman et al., 2005; Huffman, 2009; Han et al., 2013). Synthetic routes have been patented and published and SCRA receptor binding and activation data reported as a result of structure activity relationship (SAR) studies (e.g., Huffman et al., 2005; Buchler et al., 2009). With the crystal structure of the CB_1_ and CB_2_ receptors having recently been described (Hua et al., 2016; Kumar et al., 2019; Li et al., 2019; Lorenzen and Sakmar, 2019), the study of the influence of the absolute structural conformation of SCRAs on cannabinoid receptor binding and activation via agonist-initiated GPCR ligand-receptor complex induced conformational changes continues (Ibsen et al., 2017; Schoeder et al., 2018; Kumar et al., 2019). Due to variations in their molecular structure, different SCRAs may cause different conformational changes in the receptor-ligand complex conformation and as a result give rise to different cellular responses with a bias to a particular signaling pathway (e.g., G-protein coupling vs. β-arrestin recruitment). In opioid receptors, such biased signaling has been utilized to create agonists which are biased toward pathways which give rise to desired therapeutic effects rather than those which give rise to unwanted side effects (Ho et al., 2018).

In parallel to an increasing understanding of the interaction of SCRAs with cannabinoid receptors, the illicit drugs market continues to search for, produce, and supply potent CB_1_-selective or mixed selectivity SCRAs for their psychoactive effects. SCRAs have been involved in an increasing number of fatal and non-fatal intoxications globally (e.g., Shevyrin et al., 2015; Adamowicz, 2016; Gieron and Adamowicz, 2016; Adams et al., 2017; Angerer et al., 2017; EMCDDA, 2018b; Kronstrand et al., 2018). Reported adverse health effects include agitation, psychosis, anxiety, tachycardia, seizures, and hypothermia (Sherpa et al., 2015; Adams et al., 2017) and in rodent studies many of these effects have been shown to be CB_1_-receptor mediated (e.g., Banister et al., 2013, 2015b, 2016; Funada and Takebayashi-ohsawa, 2018; Wilson et al., 2019), however the influence of different signaling pathways on these effects has not yet been established.

The pharmacology of these substances has been studied in a responsive and at times, pre-emptive manner (Banister et al., 2015a, 2016, 2019b; Cannaert et al., 2016; Longworth et al., 2017; Schoeder et al., 2018; Noble et al., 2019). The origins and evolution of SCRAs from medical research tools and drug candidates to often highly potent illicit drugs have recently been extensively and elegantly reviewed (Banister and Connor, 2018a,b).

On the illicit market, SCRAs are commonly bought as crystalline solids or viscous liquids and dissolved in solvents such as acetone and alcohols for spraying or mixing with plant materials. In this way the final product mimics the visual characteristics of herbal cannabis, and such materials are then sold as herbal smoking mixtures, known collectively and colloquially as “spice” (Pützl et al., 2015; EMCDDA, 2017a). This often gives rise to heterogeneous final products, making consistent dosing difficult for users and therefore producing unpredictable effects (Moosmann et al., 2015; Frinculescu et al., 2017). There has been a considerable diversification of the products in which such compounds have been detected, including powders, e-liquids for vaping (Peace et al., 2017; Angerer et al., 2019), impregnated papers and letters (Ford and Berg, 2018), clothing and other materials. These latter forms are commonly associated with the smuggling of SCRAs into prisons where they are particularly prevalent (EMCDDA, 2017a, 2018c; National Offender Management Service, 2017; Ralphs et al., 2017; Metternich et al., 2019).

The types of SCRAs detected have evolved in response to market demand, research studies on analog potency and national and international legislative controls. Arguably the most important controls are those controlling production and supply in China, with clear evidence that this is the main point of origin for such substances. In 2015, the production and export of 116 NPS were controlled by China including 39 SCRAs (UNODC, 2015) and soon after many of these, if not all, disappeared from the market. These compounds were rapidly replaced by new analogs, and in many but not all cases, these new substances have generally been more potent (Banister and Connor, 2018b; Banister et al., 2019a,b; Noble et al., 2019). It is therefore important to develop robust and adaptable methodologies to carry out detailed analytical and pharmacological profiling of new substances as they appear on the illicit drug market and to determine the relative theoretical potency of materials containing SCRAs at all levels of the supply chain for harm reduction purposes.

**Figure 1:**
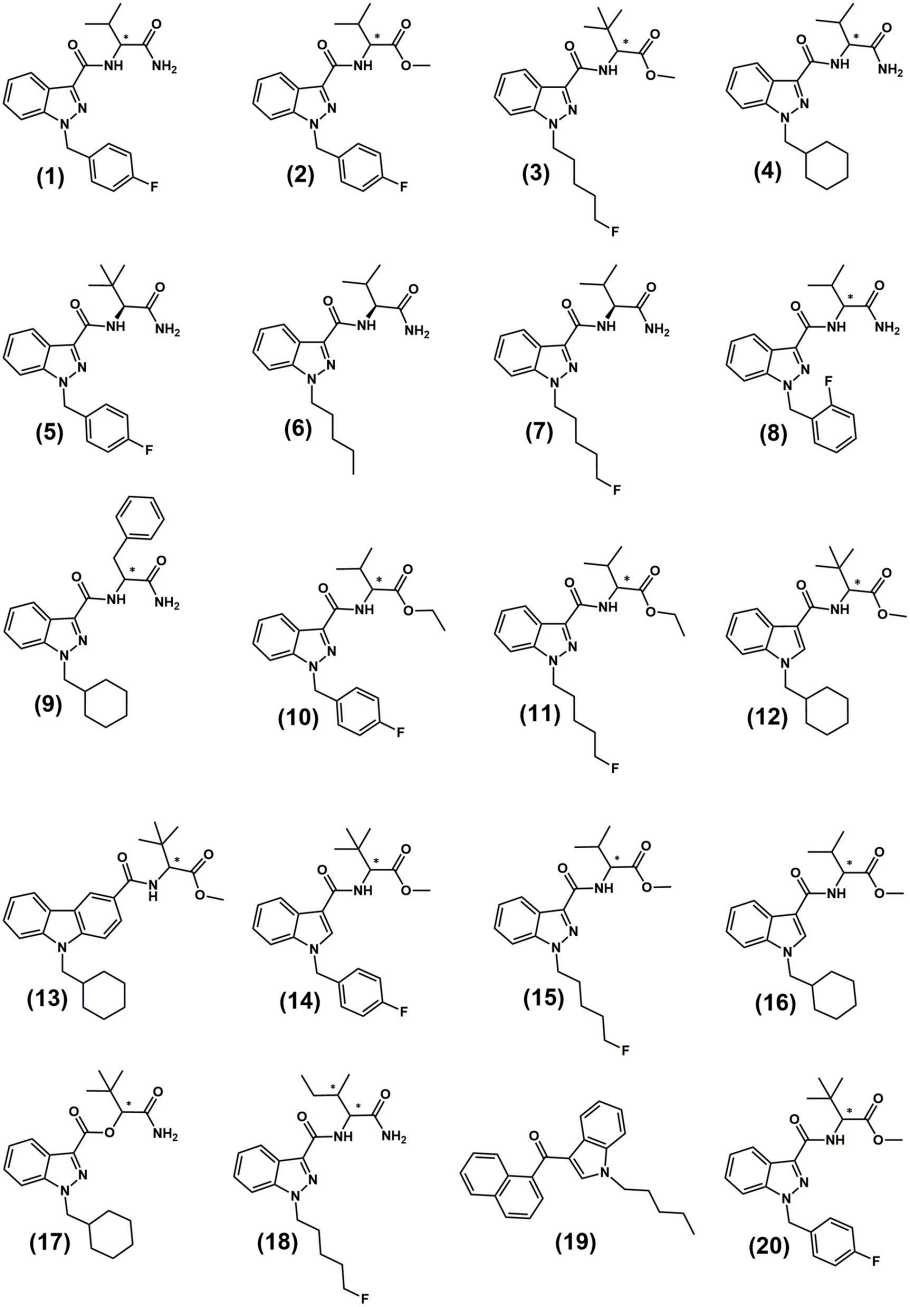
Structures of the indazole-3-carboxamide synthetic cannabinoids utilized in this study and in previous studies. **(1)** AB-FUBINACA; **(2)** AMB-FUBINACA; **(3)** 5F-MDMB-PINACA (5F-ADB); **(4)** AB-CHMINACA; **(5)** (*S*)-ADB-FUBINACA; **(6)** (*S*)-AB-PINACA; **(7)** (*S*)-5F-AB-PINACA; **(8)** AB-FUBINACA 2-fluorobenzyl isomer; **(9)** APP-CHMINACA; **(10)** EMB-FUBINACA; **(11)** 5F-EMB-PINACA **(12)** MDMB-CHMICA; **(13)** MDMB-CHMCZCA **(14)** MDMB-FUBICA; **(15)** 5F-AMB; **(16)** AMB-CHMICA (MMB-CHMICA); **(17)** ADB-CHMINACA (MAB-CHMICA); **(18)** (*1S,2S*)-5F-ADB-PINACA isomer 2. **(19)** JWH-018; **(20)** MDMB-FUBINACA. ^*^Indicates the position of the chiral centre.

In recent years, SCRAs with acylindole and acylindazole scaffolds have become amongst the most prevalent and potent available on illicit markets. Relevant chemical structures discussed in this study are provided in Figure 1 and numbers in bold parentheses, e.g., **(1)**, refer to these structures throughout the text. The most common of these compounds in recent years have been some of the many analogs developed by Pfizer Global Research & Development and patented in 2009 as potential analgesics (Buchler et al., 2009; Banister et al., 2015a; Banister and Connor, 2018a). The valine- and *tert*-leucine derived indazole-3-carboxamide analogs are chiral molecules and can therefore theoretically be present in two enantiomeric forms. Their relative prevalence will depend upon the source of the precursor chemicals used and their enantiopurity. Amongst a wide array of other compounds, the original Pfizer patent (Buchler et al., 2009) utilized L-*tert*-leucine methyl ester, L-valinamide and L-leucinamide to give (*S)*-enantiomer products [e.g., (*S*)-AB-FUBINACA **(1)** (*N*-(*2S*)-(1-amino-3-methyl-1-oxobutan-2-yl)-1-(4-fluorobenzyl)-*1H*-indazole-3-carboxamide], (*S*)-AMB-FUBINACA **(2)** methyl (*2S*)-2-(1-(4-fluorobenzyl)-*1H*-indazole-3-carboxamido)-3-methylbutanoate, (*S*)-AB-CHMINACA **(4)** (*N*-(*2S*)-(1-amino-3-methyl-1-oxobutan-2-yl)-1-(cyclohexylmethyl)-*1H*-indazole-3-carboxamide) and (*S*)-ADB-FUBINACA **(5)** (*N*-(*2S*)-(1-amino-3,3-dimethyl-1-oxobutan-2-yl)-1-(4-fluorobenzyl)-*1H*-indazole-3-carboxamide)). No assessment of enantiopurity of precursors or products was reported and no synthesis methods or pharmacological data for the possible (*R*)-enantiomers were published (Buchler et al., 2009). This group of compounds also lead to the arrival of some of the most potent SCRAs to be seen on the illicit market, including 5F-MDMB-PINACA (**(3)**, more commonly referred to as 5F-ADB) and MDMB-FUBINACA **(20)**.

There is a growing interest in the chromatographic separation of the increasing diversity of chiral NPS (as well as more traditional drugs of abuse such as amphetamine and methamphetamine), to gain additional information on the synthetic routes used in their manufacture and to indicate their inherent potency and efficacy (Taschwer et al., 2017; Kadkhodaei et al., 2018; Silva et al., 2018). There are opportunities to add chiral profiling to existing drug profiling methodologies if there is reproducible variation in the chiral profiles of precursor materials, even if the absolute differences are small. Chiral profiling can also aid in the assessment of a seized material's potency and therefore in risk assessment and harm reduction strategies. Using a Diacel Chiralpak® AZ-3R column, Doi et al. (2016, 2018) reported the reverse phase HPLC separation of a number of carboxamide-type SCRA enantiomers (5F-AB-PINACA **(7)** (*N*-(1-amino-3-methyl-1-oxo-2-butanyl)-1-(5-fluoropentyl)-1H-indazole-3-carboxamide), AB-FUBINACA 2-fluorobenzyl isomer **(8)** (*N*-(1-amino-3-methyl-1-oxobutan-2-yl)-1-(2-fluorobenzyl)-1H-indazole-3-carboxamide), APP-CHMINACA **(9)** (*N*-(1-amino-1-oxo-3-phenylpropan-2-yl)-1-(cyclohexylmethyl)-1H-indazole-3-carboxamide), EMB-FUBINACA **(10)** (ethyl 2-(1-(4-fluorobenzyl)-1H-indazole-3-carboxamido-3-methylbutanoate), 5F-EMB-PINACA **(11)** (ethyl 2(-1-(5-fluoropentyl)-1*H*-indazole-3-carboxamido)-3-methylbutanoate), MDMB-FUBICA **(14)** (methyl 2-(1-(4-fluorobenzyl)-*1H*-indole-3-carboxamido)-3,3-dimethylbutanoate) and 5F-AMB **(15)** (methyl 2-(1-(5-fluoropentyl)-*1H*-indazole-3-carbamido)-3-methylbutanoate). The separation of MDMB-CHMICA **(12)** (methyl-2-(1-(cyclohexylmethyl)-*1H*-indole-3-carboxamido-3,3-dimethyl-butanoate) and MDMB-CHMCZCA **(13)** (methyl 2-(9-(cyclohexylmethyl)-9*H*-carbazole-3carboxamido)-3-methylbutanoate) enantiomers using a Chiralpak® IA-3 column in normal phase mode has also been reported (Andernach et al., 2016; Weber et al., 2016).

This study describes the enantiospecific synthesis and chemical and pharmacological characterization of four indazole-3-carboxamide SCRA enantiomer pairs (AB-FUBINACA **(1)**, AMB-FUBINACA **(2)** 5F-MDMB-PINACA **(3)** and AB-CHMINACA **(4)**). All four SCRAs have been detected in seized samples and two, AMB-FUBINACA and 5F-MDMB-PINACA, have been amongst the most prevalent compounds detected in Europe, particularly in the UK, and the United States for several years (EMCDDA, 2017b, 2018a,b; Centre for Forensic Science Education, 2018; DEA, 2018a,b). In September 2018, a ban on the production and export of seven of the most potent SCRAs, including AMB-FUBINACA **(2)** and 5F-MDMB-PINACA **(3)** was announced by the Chinese Government^1^ (UNODC, 2018b). This could lead, as previously observed with the earlier 39 SCRAs controlled in a similar way in 2015, to their rapid disappearance from the illicit market. In the same way they are likely to be replaced either by completely new analogs or by similar chiral compounds with small structural alterations, e.g., their azaindole analogs, or modifications to the *N*-alkyl/benzyl chain where the length and/or position of the substituent (normally fluorine) is varied. Such compounds include the recently reported chiral SCRAs, 4F-MDMB-BINACA (methyl 2-((1-(4-fluorobutyl)indazole-3-carbonyl)amino)-3,3- dimethyl-butanoate), 5F-MDMB-PICA (Centre for Forensic Science Education, 2019), 5F-AB-P7AICA (*N*-(1-amino-3-methyl-1-oxobutan-2-yl)-1-(5-fluoropentyl)-1*H*-pyrrolo[2,3-b]pyridine-3-carboxamide) (Adebar Project, 2018) and APP-BINACA (*N*-(1-amino-1-oxo-3-phenylpropan-2-yl)-1-butyl-*1H*-indazole-3-carboxamide) (EMCDDA, 2019). The rapid and efficient synthesis of enantiopure reference standards and the development of robust separation methods for their ongoing monitoring and evaluation is therefore required to facilitate full analytical and pharmacological assessment. Although there has to date been relatively little variation in the SCRA chiral profiles reported in the literature, the number of samples that have been tested is small and there is not yet an understanding of the variability and reproducibility of the small but potentially discriminatory differences between batches and/or precursor suppliers. An underlying understanding of the chiral separation mechanisms provides the ability to adapt methods rapidly to the inevitable arrival of new chiral SCRAs or the resurgence of others not yet under such control.

This study also reports the relative potency and efficacy of the SCRAs described using previously reported stable cell-based assays that monitor CB_1_ and CB_2_ receptor activation and are based on the recruitment of the cytosolic protein, β-arrestin 2 (βarr2) to the activated receptor. The methodology has previously been applied to both pure compounds and toxicological samples and has recently been fully validated (Cannaert et al., 2016, 2017, 2018, 2019a,b; Noble et al., 2019) and will be used to gain further insights into SCRA pharmacology in the light of recent studies (e.g., Kumar et al., 2019). A comparative approach for the assessment of street and prison seized SCRA samples, combining the determination of SCRA concentrations and where possible including the elucidation of chiral profiles considers the concept of “estimated intrinsic potency” of a sample is introduced. Such an approach could provide a more logical way to assess the potential for harm and to communicate risk effectively in a context where individual SCRA compounds vary widely in structure and potency and vary geographically and temporally.

## Materials and Methods

### Chemicals

**Figure 2:**
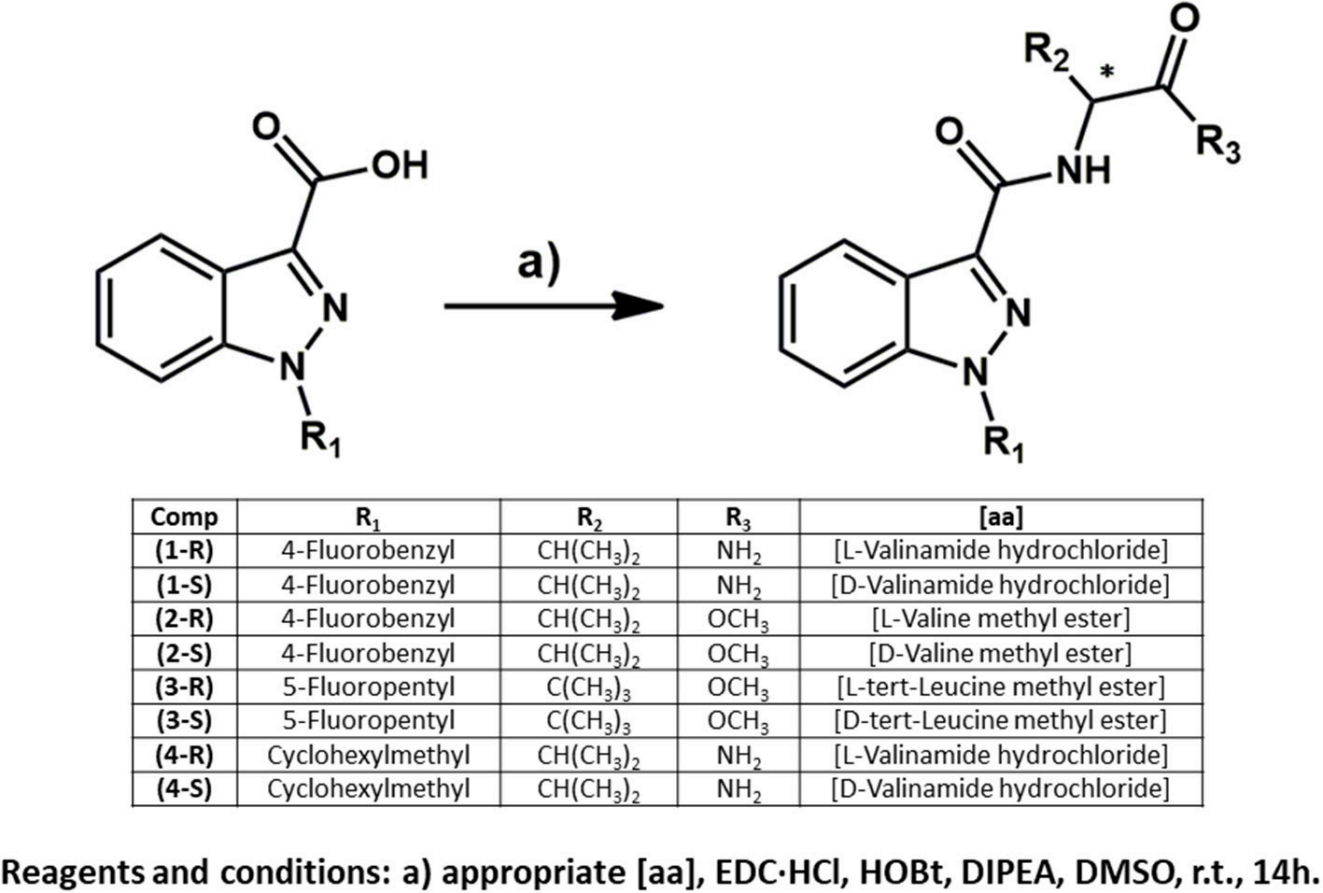
Enantiospecific synthesis of eight indazole-3-carboxamide synthetic cannabinoids. Compound numbering relates to that provided in Figure 1.

Reagents for the synthesis of the enantiopure reference standards (see Figure 2) were obtained from Sigma-Aldrich, Gillingham, UK; Alfa Aesar, Heysham, Lancashire, UK; and Fluorochem Limited, Hadfield, UK, and were used without further purification. Custom synthesized D-*tert*-leucine was supplied by Carbosynth, UK. Solvents used in the synthesis (including deuterated solvents) were acquired from Sigma-Aldrich or Thermo Fisher Scientific, Loughborough, UK. All solvents used for HPLC and GC-MS analysis were HPLC grade and supplied by Fisher, UK. Ultra-high purity water (18 MΩcm^−1^) was obtained using a Milli-Q water purification system (Merck, UK).

#### Enantiospecific Synthesis

The *N*-substituted indazole-3-carboxylic acids were synthesized following procedures previously reported in the literature (Banister et al., 2015a, 2016) or adaptations of them (Figure 2). The synthesis of AB-CHMINACA was based on the synthesis of AMB-CHMINACA with valinamide being substituted for the valine methyl ester (see Figure 2) and the synthesis optimized by the addition of another equivalent of potassium tert-butoxide and bromomethyl cyclohexane and the reaction being heated to a reflux for 18 h instead of being stirred at room temperature for 48 h as described in the original method.

#### Reference Standard Characterization

##### Nuclear magnetic resonance (NMR) spectroscopy

NMR spectroscopy for the 5F-MDMB-PINACA, AB-FUBINACA and AMB-FUBINACA enantiomers was performed using a JEOL ECS-400 NMR spectrometer (JEOL, Tokyo, Japan) operating at 400 MHz. ^1^H-NMR (10 mg/mL in CDCl_3_), ^13^C-NMR (20 mg/mL in CDC_L3_). NMR spectroscopy for the AB-CHMINACA enantiomers was performed using a Bruker AVANCE III HD 500 MHz spectrometer (Bruker, Billerica, MA, USA) running under TopSpin v.3.2.5 and equipped with a QCI-F cryo-probe at a sample compartment temperature of 25°C. Samples were prepared in CDCl_3_ (~10 mg/mL).

##### Gas chromatography-mass spectrometry (GC-MS)

GC-MS analysis of reference standards (100–1,000 μg/mL) and seized sample extracts was carried out using a 7820A gas chromatograph coupled to a 5977E mass spectrometer (Agilent Technologies, Santa Clara, CA, USA). Injection mode: 1 μL sample injection and 20:1 split (and a 5:1 split for trace component screening), injection port temperature: 200°C, carrier gas: He, flow: 1 mL/min. Column: DB-1MS, 0.33 μm, 0.2 mm × 25 m (Agilent Technologies) (EMCDDA, 2017d). GC oven: 80°C held for 3 min; 40°C/min to 300°C held for 3.5 min; transfer line: 295°C. The mass spectrometer operated in electron ionization (EI) mode. Ionization conditions: 70 eV in full scan mode (50–550 amu), ion source: 230°C, quadrupole: 150°C.

##### High performance liquid chromatography with photodiode array detection (HPLC-PDA)

Initial chiral column screening was carried out using an Agilent Infinity II HPLC system with photodiode array detection (PDA). The selectivity of Lux® Amylose-1, Lux® Cellulose-1-4 columns (all 5 μm, 4.6 × 100 mm) and a Lux® i-Cellulose-5 column (5 μm, 4.6 × 150 mm column) from Phenomenex, UK, were assessed by triplicate 5μL injections of a 0.2mg/mL SCRA racemic mixture standards using 50:50 MilliQ H_2_O (A) : ACN (B) with a flow rate of 1.0 mL/min. The two most selective chiral stationary phases (CSPs) were identified and optimized column formats selected (Lux® Amylose-1, 3 μm, 2.1 × 150 mm and Lux® i-Cellulose-5, 3 μm, 2.1 × 150 mm). To assess their chromatographic performance in more detail, triplicate 1 μL injections of a 200 μg/mL reference standard solution in both acetonitrile and methanol were made in isocratic mode at 45:55, 50:50, and 55:45 (A:B) with a flow rate of 0.2 mL/min. After optimization and after assessing the SCRAs known to be present in the seized samples to be analyzed, the Lux® Amylose-1 column was used for the analysis of seized samples.

##### High performance liquid chromatography-photodiode array-quadrupole time of flight mass spectrometry (HPLC-PDA-QToF-MS/MS)

High resolution mass spectrometry (HRMS*)* analysis of the synthesized reference standards and seized samples was performed using an Acquity UPLC® instrument consisting of a binary pump, autosampler (held at 4°C), vacuum degasser and column oven (held at 30°C), coupled to a Xevo-QToF-MS/MS (Waters Corporation, Milford, MA, USA). Mobile phases were (A) LC-MS grade water with 0.1% formic acid and (B) acetonitrile with 0.1% formic acid. Flow rate was 0.2 mL/min and 1 μL of sample was injected onto a Lux Amylose-1, 3 μm, 2.1 × 150 mm, column. The QToF was operated in positive ionization mode with a source temperature of 120°C, a desolvation temperature at 500°C and a capillary voltage at 2.25 kV. ToF-MS analysis for the high-resolution determination of molecular mass and calculation of mass error (ppm) was carried out with a collision energy at 6 V. MS^e^ aquisition was carried out using collision energies ranging from 6 to 28 V. Once QTOF-MS, and MS^e^ data were processed, MS/MS data acquisition was utilized for selected parent ion accurate mass data to provide accurate product ion data. UV spectra (200–400 nm) were collected using an Acquity® PDA detector.

### Cannabinoid Receptor Assay

To assess the biological activity of the synthesized reference compounds, live cell-based reporter assays that monitor protein-protein interactions via NanoLuc Binary Technology were used as described previously (Cannaert et al., 2018). The receptor activation is evaluated via the interaction between βarr2, a cytosolic protein, and the GPCRs CB_1_ and CB_2_. Both βarr2 and CB_1_/CB_2_ are fused to an inactive part of nanoluciferase. When CB_1_ or CB_2_ are activated by a ligand, βarr2 is recruited to the receptor, allowing interaction of the complementary nanoluciferase subunits, yielding a functional enzyme that generates a bioluminescent signal in the presence of the substrate furimazine. Curve fitting and statistical analyses were performed using GraphPad Prism software (San Diego, CA, USA). The results are represented as mean area under the curve (AUC) ± standard error of mean (SEM) with at least seven replicates for each data point (obtained in three independent experiments). Curve fitting of concentration-effect curves via non-linear regression (four parameter logistic fit) was employed to determine EC_50_ (measure of potency) and E_max_ values (measure of efficacy). The E_max_ values are normalized to the E_max_ value of JWH-018 (100%).

### Seized Samples

Suspected SCRA samples, all in clear plastic “Snap bags” containing herbal material purported to be “spice” were seized by Greater Manchester Police (GMP) in Piccadilly Gardens, Manchester, UK between 21 March 2017 and 15 January 2018. They are provided to this study *via* the MANchester DRug Analysis and Knowledge Exchange (MANDRAKE) partnership between GMP and Manchester Metropolitan University. All bulk samples had previously undergone preliminary qualitative analysis prior to inclusion in this study.

### Seized Sample Analysis

#### Qualitative and Quantitative Achiral GC-MS Analysis

For each SCRA sample, three replicate aliquots of approximately 10 mg herbal material were accurately weighed out. Each 10 mg aliquot was extracted sequentially using 3 × 1 mL of 75:25 dichloromethane:methanol with 10 min ultrasonication, after which the three extracts were combined. A 200 μL aliquot of this extract was diluted to 1 mL using an internal standard (tridecane) solution in 75:25 dichloromethane:methanol to give a final internal standard concentration of 35.5 μg/mL. For trace component screening, 50 mg of sample was extracted by sonication for 10 min with 1 mL methanol. The four indazole-3-carboxamide SCRAs for which in house reference standards were synthesized were identified by comparison of retention times and mass spectra between the seized samples and the reference standards. Confirmation of analyte identity was made by reference to the Cayman spectral library (version 12212018) and SWGDRUG spectral library (version 3.3) with a minimum reverse match score of 800 required for identification. SCRA concentrations are provided as an average concentration (mg/g) and range, reflecting the heterogeneous nature of the herbal materials (the samples were purposefully not homogenized to retain this information). Other SCRAs detected, for which reference standards were not available in house, were tentatively identified by reference to the mass spectral libraries with corroboration provided by HPLC-QToF-MS/MS spectra and reference to previously published HRMS spectra. A series of calibration standards were prepared and used to generate non-linear calibration curves (*R*^2^ > 0.99) for the quantitation of 5F-MDMB-PINACA and AMB-FUBINACA in seized herbal samples (10 to 200 μg/mL, each with 35 μg/mL tridecane as internal standard). The precision of the instrument and accuracy of the calibration curve was verified by the replicate (*n* = 12) analysis of an independent calibration check standard (30 μg/mL), yielding a coefficient of variance (CV) of 3.6 and 3.0% and bias of +3.2 and −4.6% for 5F-MDMB-PINACA and AMB-FUBINACA, respectively. The estimated concentrations of the SCRAs present in seized samples for which in house reference standards were not available were calculated using the detector response for AMB-FUBINACA and these are clearly noted in the text and figures as semi-quantitative results.

#### Qualitative Chiral UPLC®-PDA-QToF-MS/MS Analysis

Approximately 50 mg of seized sample was accurately weighed out and sequentially extracted using 3 × 1 mL of methanol. The extracts were combined and filtered using a 0.45 μm syringe filter prior to transferring a 1 mL aliquot to a 2 mL sample vial. Further dilutions (10x, 100x, and 1,000x) were prepared to establish the linearity of the PDA and MS/MS detector response. Enantiomeric ratios were determined by integration of the UV chromatogram at 210 nm.

## Results and Discussion

In a drug misuse and harm reduction setting, understanding the relative potency (and therefore potential effects and harms) of different drug batches or drug presentations is of critical importance. In legal terms, for a seized bulk drug or drug product, the identification of the substance(s) present will be of paramount importance to determine legality. In addition, any information that may facilitate batch linkage and source identification is also worthy of study. The relative potency of a particular SCRA containing product will be related to (i) the type(s) of SCRAs present within the sample and (ii) the concentrations of those SCRAs.

### Synthesis

(*S)*- and (*R*)-enantiomers of four indazole-3-carboxamide SCRAs (AB-FUBINACA **(1)**, AMB-FUBINACA **(2)**, 5F-MDMB-PINACA **(3)** and AB-CHMINACA **(4)**) were successfully synthesized. With the exception of 5F-MDMB-PINACA, (*R*)-enantiomers of these SCRAs are not currently commercially available. All enantiopure standards were calculated to have an enantiopurity of >99.8% by GC-MS and HPLC-PDA, NMR and HRMS data. Full characterization data (NMR, GC-MS, HPLC-QToF-MS/MS, UV) for all enantiopure reference standards were similar between enantiomer pairs and in agreement with their expected structures and are provided in the Supplementary Information.

### Activity-Based Receptor Bioassay Results

**Figure 3:**
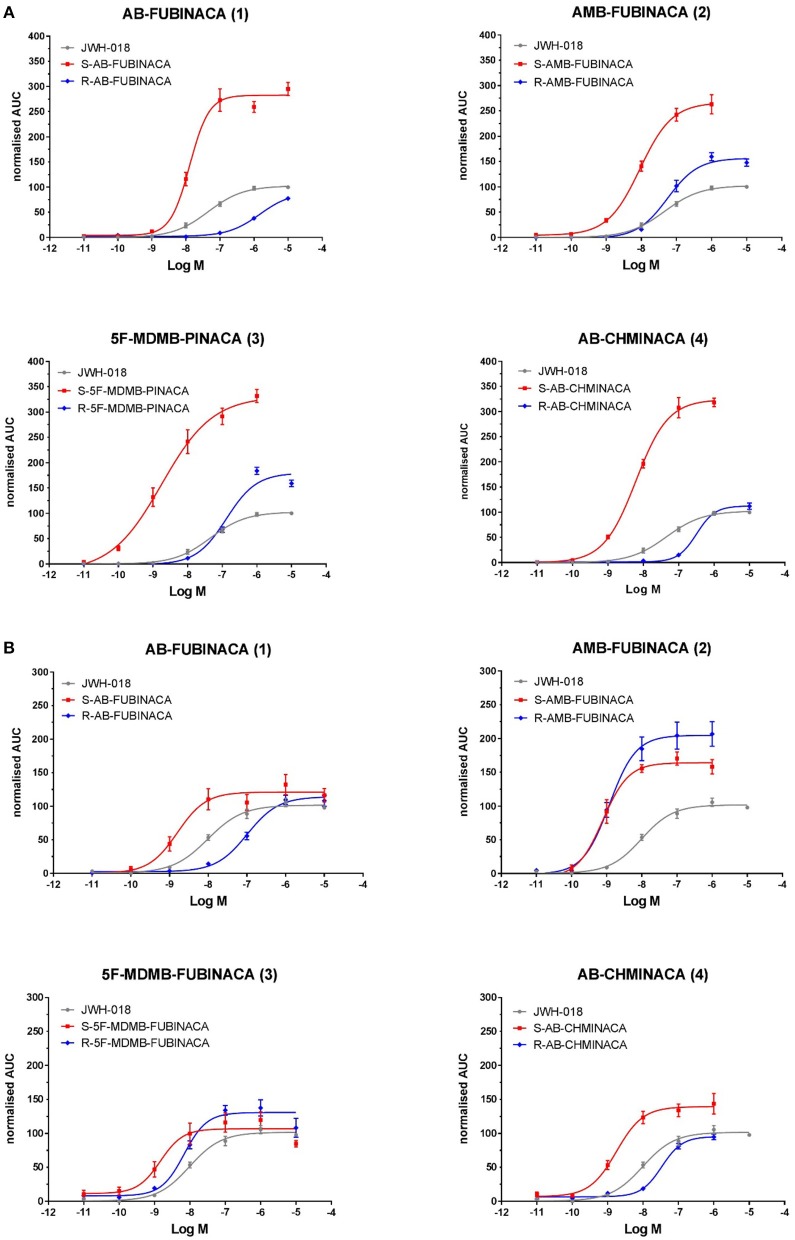
EC_50_ curves of indazole-3-carboxamide synthetic cannabinoids for **(A)** CB_1_ and **(B)** CB_2_ receptors. Each data point represents the mean value and error bars represent the standard error of the mean (SEM) for at least seven replicates (obtained in 3 independent experiments). Compound numbering relates to that provided in Figure 1.

We compared the potencies of all synthesized reference standards at CB_1_ and CB_2_ receptors (Table 1, Figure 3). All eight compounds (four enantiomer pairs) activated CB_1_ and CB_2_ receptors as would be expected from previous studies on the (*S*)-enantiomers of these compounds. (*S*)-AB-FUBINACA had previously been analyzed using the same bioassay (Noble et al., 2019) and results between the two analyses were consistent as were the data for the positive control compound (JWH-018). All (*S*)-enantiomers had higher potency (EC_50_) and efficacy (E_max_) values at CB_1_ than the control JWH-018 (2.5–3.4 times E_max_), which is commonly described as a full agonist, as did the (*R*)-enantiomers of 5F-MDMB-PINACA and AMB-FUBINACA (1.5 and 1.8 times JWH-018 E_max_), suggesting that these compounds strongly and/or stably recruit βarr2 to CB_1_ in comparison to JWH-018. The high CB_1_ efficacy values of the (S)-enantiomers compared to JWH-018 obtained from this assay, might suggest that the assay ceiling has not yet been met. This could be tested in future studies by determining the relative efficacy of the high efficacy SCRAs before and after depleting the receptor reserve with an irreversible CB1 antagonist, such as AM6544 (Sachdev et al., 2018). For each enantiomer pair, the (*S*)-enantiomer was considerably more potent than the corresponding (*R*)-enantiomer, in agreement with the only other previous study for related indazole-3-carboxamides (Doi et al., 2018). The difference in potency between enantiomer pairs was less pronounced for CB_2_ than CB_1_ as has previously been observed previously. For CB_1_ the differences ranged from 6.11- to 114-fold depending on the enantiomer pair, and for CB_2_ 1.55- to 63.5-fold. The relative potency of the enantiomers to the CB_2_ receptor is affected by structural features, the difference being more pronounced for compounds with an amine moiety (AB-FUBINACA and AB-CHMINACA) than those with an ester moiety (AMB-FUBINACA and 5F-MDMB-PINACA).

**Table 1:** Half-maximal effective concentration (EC_50_) and maximal response (E_max_) compared to JWH-018 (control), the tested indazole-3-carboxamide synthetic cannabinoids in this study compared to previously reported data.

**Compound**	**CB**_****1****_		**CB**_****2****_		**CB_**1**_/CB_**2**_ ratio**	**R/S ratio CB_**1**_**	**R/S ratio CB_**2**_**	
	**EC_**50**_ (nM) (+/– 95% CI)**	**E_**max**_ (%) (+/– 95% CI)**	**EC_**50**_ (nM) (+/– 95% CI)**	**E_**max**_ (%) (+/– 95% CI)**				
**RESULTS OBTAINED USING ACTIVITY-BASED RECEPTOR BIOASSAY**								
JWH-018 (control)	45.1 (32.4–62.9)	102.6 (97.1–109)	9.71 (6.52–14.8)	102 (95.0–109)	4.64	–	–	This study
(*S*)-AB-FUBINACA **(1)**	12.9 (10.1–18.1)	283 (265–302)	1.60 (0.67–3.65)	121 (107–137)	8.06	114	63.5	
(*R*)-AB-FUBINACA **(1)**	1480 (977–4770)	90.2 (78.6–127)	102 (68.4–153)	114 (104–127)	14.5			
(*S*)-AMB-FUBINACA **(2)**	9.11 (6.07–14.1)	267 (247–293)	0.77 (0.43–1.24)	161 (152–178)	11.8	6.13	1.55	
(*R*)-AMB-FUBINACA **(2)**	55.8 (39.3–78.1)	154 (144–164)	1.19 (0.65–2.29)	205 (185–228)	46.9			
(*S*)-5F-MDMB-PINACA **(3)**	1.78 (0.72–4.11)	331 (293–406)	1.46 (0.48–4.15)	107 (94.2–120)	1.21	73.6	4.71	
(*R*)-5F-MDMB-PINACA **(3)**	131 (98.6–174)	180 (170–190)	6.87 (4.08–10.0)	131 (121–141)	19.1			
(*S*)-AB-CHMINACA **(4)**	6.16 (4.49–8.35)	324 (307–344)	1.75 (1.06–3.34)	139 (127–154)	3.52	51.8	19.2	
(*R*)-AB-CHMINACA **(4)**	319 (242–410)	113 (107–119)	33.6 (23.9–46.0)	95.3 (88.8–102)	9.50			
JWH-018 (control)	41.0 (33.4–50.3)	99.3 (95.8–102.8)	12.3 (9.89–15.4)	104 (100–108)	3.33	–	–	Noble et al., 2019
(*S*)-AB-FUBINACA **(1)**	15.6 (10.4–23.2)	324 (302–346)	1.78 (0.80–3.93)	107 (92.7–120)	9.21	–	–	
(*S*)-ADB-FUBINACA **(5)**	0.69 (0.46–1.02)	339 (307–370)	0.59 (0.43–0.80)	135 (124–145)	1.17	–	–	
(*S*)-AB-PINACA **(6)**	18.5 (13.4–25.6)	288 (272–304)	2.77 (1.31–5.83)	144 (125–162)	6.68	–	–	
(*S*)-5F-AB-PINACA **(7)**	65.5 (40.7–106)	268 (243–293)	7.06 (4.18–12.0)	173 (154–192)	9.28	–	–	
JWH-018 (control)	23.9 (18.3–31.6)	–	6.80 (3.29–13.8)	–	3.51			Cannaert et al., 2017
ADB-CHMINACA **(17)**	1.49 (0.69–2.61)	–	2.20 (1.00–4.30)	–	0.68			
**RESULTS OBTAINED USING [**^**35**^**S]GTPγS BINDING ASSAY**								
(*S*)-AB-FUBINACA 2-fluorobenzyl isomer **(8)**	2.92	–	14.1	–	1.20	4.82	0.257	Doi et al., 2018
(*R*)-AB-FUBINACA 2-fluorobenzyl isomer **(8)**	2.44	–	0.627	–	22.5			
(*S*)-APP-CHMINACA **(9)**	251	–	33700	–	31.0	134	60.6	
(*R*)-APP-CHMINACA **(9)**	8.09	–	490	–	68.8			
(*S*)-EMB-FUBINACA **(10)**	0.458	–	30.7	–	0.214	67.0	0.403	
(*R*)-EMB-FUBINACA **(10)**	2.14	–	0.863	–	35.6			
(*S*)-5F-EMB-PINACA **(11)**	4.96	–	35.9	–	0.718	7.24	0.243	
(*R*)-5F-EMB-PINACA **(11)**	6.91	–	1.68	–	21.4			

With an EC_50_ of 1.78 nM (Table 1), (*S*)-5F-MDMB-PINACA **(3)** has the highest potency at CB_1_ of all the compounds tested in this study, followed by the (*S*)-enantiomers of AMB-FUBINACA >AB-CHMINACA >AB-FUBINACA. The potency of (*S*)-5F-MDMB-PINACA is second only to (*S*)-ADB-FUBINACA previously tested using the same bioassay under the same conditions (Noble et al., 2019). (*S*)-5F-MDMB-PINACA was four times more potent at CB_1_ than (S)-AMB-FUBINACA **(4)**, consistent with previously reported data using the FLIPR assay which measures changes in membrane potential (Banister et al., 2015b).

(*R*)-AMB-FUBINACA tested in this study was more potent than any other (*R*)-enantiomer in this study and was more potent than both the indazole-3-carboxamides, (*S*)-JWH-018 (control, this study) and (*S*)-AB-PINACA **(6)** and the indole-3-carboxamides 5F-AMB-PICA and 5F-PY-PICA analyzed previously using the same assay under the same conditions (Noble et al., 2019). The latter compound has been found to have little or no CB_1_ activity, a finding confirmed by Banister et al. (2019a).

AB-FUBINACA **(1)**, with a terminal amide moiety (valinamide), and AMB-FUBINACA **(2)**, with a terminal methyl ester (valinate) moiety, are otherwise structurally identical, both having a para-fluorobenzyl moiety linked to the indazole core. The (*S*)-enantiomers have a similar potency at CB_1_ but exhibited a marked difference in the EC_50_ ratio between *(S)* and *(R)* enantiomers between the compounds. For AMB-FUBINACA **(2)** the (*S*)-enantiomer was 6.1 times as potent as the (*R*)-enantiomer whilst for AB-FUBINACA **(3)** the (*S*)-enantiomer was >100 times more potent than the (*R*)-enantiomer. This difference may be explained by differential interactions between the enantiomer pairs and the CB_1_ receptor, at the valinamide and valinate moieties.

Using the structurally similar and highly potent SCRA, MDMB-FUBINACA **(20)**, Kumar et al. (2019) demonstrated that the indazole group and *p*-fluorobenzyl moiety is most likely involved in hydrophobic interactions with receptor elements, the latter within a conserved docking region (described as a narrow side pocket). It is suggested that this narrow pocket is an important region influencing ligand affinity rather than activation whilst the indazole region influences receptor activation and therefore more clearly affects potency. Both observations are supported by Schoeder et al. (2018) and previous studies who noted that indazoles are more potent than indoles but that replacing the *p*-fluorobenzyl moiety with a pentyl chain gives compounds with similar receptor affinity and similar potency. It is suggested that the ester moiety of MDMB-FUBINACA **(20)**, which is also present in AMB-FUBINACA **(2)**, then forms polar interactions with the H178^2.65^ region of the receptor (Kumar et al., 2019). AB-FUBINACA **(1)** has an amide moiety in this position rather than an ester, which could also create polar interactions and has a similar potency to AMB-FUBINACA **(2)** in this study. This suggests that as with the fluorobenzyl/fluoro-alkyl chain in the “narrow side pocket” region, the methyl ester/amide moiety may influence ligand affinity rather than receptor activation. The “bulky” tert-butyl moiety of MDMB-FUBINACA **(2)** produces a more potent compound than the closely related AMB-FUBINACA **(3)** suggesting that it is this region, when all other structural features are in place to ensure ligand affinity, which is involved in receptor activation and therefore influences potency. AB-FUBINACA **(1)** and AMB-FUBINACA **(2)** both have a dimethyl group in this region and both have an indazole core and so have similar potency. Chirality will affect the relative positions of the functional groups within this region and most likely, as a result, will affect their interaction with receptor binding and activation sites. Potency is more conserved for the (*R*)-AMB-FUBINACA than for (*R*)-AB-FUBINACA and so the amide moiety may be affecting both ligand affinity and receptor activation in this spatial arrangement to a greater degree than the ester moiety. In this study 5F-MDMB-PINACA **(3)** is the only compound with, like MDMB-FUBINACA **(20)**, a tert-butyl and ester moiety. The (*S*)-enantiomer is the most potent compound studied here and the (*R*)-enantiomer is 70 times less potent. It appears that retention of potency in the (*R*)- enantiomer is greater if there is a valinate methyl ester configuration rather than a valinamide, leucinamide or *tert*-leucine methyl ester configuration.

### Chiral HPLC Separation Method Development

The underlying mechanisms of the chiral selectivity of a chiral stationary phase (CSP) have been reviewed by Berthod (2010). Selectivity is believed to occur as a result of a differential three-point interaction between the enantiomers and the CSP. Chiral separation is based on the formation of intermediate diastereoisomeric complexes between the CSP and the enantiomers in the racemic mixture. One enantiomer will have a three-point interaction with the CSP and the other a two-point interaction based on the spatial arrangement of the molecule around the chiral center. As the interactions are still relatively poorly understood, the prediction of the best CSP for a given racemate normally relies on past experience of similar tested compounds or trial and error. As the SCRAs are neutral molecules, the variation of the pH at which the separation occurs has little influence on chromatographic selectivity. The results of the initial column screening tests are shown in Table 2. The Lux® Amylose-1 (Amylose tris(3,5-dimethylphenylcarbamate)) and Lux® i-Cellulose-5 (Cellulose tris(3,5-dichloro-phenylcarbamate)) columns provided the greatest selectivity for the SCRAs tested (AB-CHMINACA **(4)** enantiomer standards were not available for the initial CSP screening experiments). The dimensions of these columns were optimized and 3 μm, 2.1 × 150 mm columns containing each phase were selected for further evaluation and optimization in reverse phase mode. The same CSP in a different column format, CHIRALPAK® IA-3 (3 μm, 4.6 × 250 mm; Amylose *tris*(3,5-dimethylphenylcarbamate) immobilized on 3 μm silica gel), has previously been used to separate MDMB-CHMICA **(12)** and MDMB-CHMCZCA **(13)** enantiomers in normal phase mode (Andernach et al., 2016; Weber et al., 2016). Doi et al. (2016, 2018) also reported on the use of an amylose-based chiral selector CHIRALPAK® AZ-3R (3.0 μm, 2.1 × 150 mm; amylose *tris*(3-chloro-4-methylcarbamate) column) in reverse phase mode to separate carboxamide-type SCRAs.

**Table 2:** Preliminary Screening of Phenomenex Lux® Chiral HPLC columns for indazole-3-carboxamide synthetic cannabinoid enantiomer separation.

**Chiral HPLC column**	**Coated or immobilized on silica gel?**	**Chiral stationary phase (CSP)**	**Resolution between Enantiomer pairs using 50:50 MilliQ:ACN isocratic method** ^****a****^		
			**AB-FUBINACA**	**5F-MDMB-PINACA**	**AMB-FUBINACA**
Lux® Amylose-1, 100 × 4.6 mm, 5 μm	Coated	Amylose *tris*(3,5-dimethylphenylcarbamate)	3.20	2.46	1.21
Lux® Cellulose-1, 100 × 4.6 mm, 5 μm		Cellulose *tris*(3,5-dimethylphenylcarbamate)	0.59	0.49	0.00
Lux® Cellulose-2, 100 × 4.6 mm, 5 μm		Cellulose *tris*(3-chloro-4-methylphenylcarbamate)	3.00	0.57	0.93
Lux® Cellulose-3, 100 × 4.6 mm, 5 μm		Cellulose *tris*(4-methylbenzoate)	0.00	0.13	0.11
Lux® Cellulose-4, 100 × 4.6 mm, 5 μm		Cellulose *tris*(4-chloro-3-methylphenylcarbamate)	2.69	0.00	1.45
Lux® i-Cellulose-5, 150 × 4.6 mm, 5 μm	immobilized	Cellulose *tris*(3,5-dichlorophenylcarbamate)	3.55	1.51	0.62

For the columns selected for further study, there was no difference in chromatographic performance as a result of using either methanol or acetonitrile as an injection solvent. The addition of 0.1% formic acid to the mobile phase solvents had negligible effect on selectivity and chromatographic performance, meaning that the method could be transferred to an HPLC-PDA-QToF-MS/MS system without further adaptation. Preliminary investigations suggest that both columns lose their selectivity when methanol is used instead of acetonitrile as a mobile phase component.

**Figure 4:**
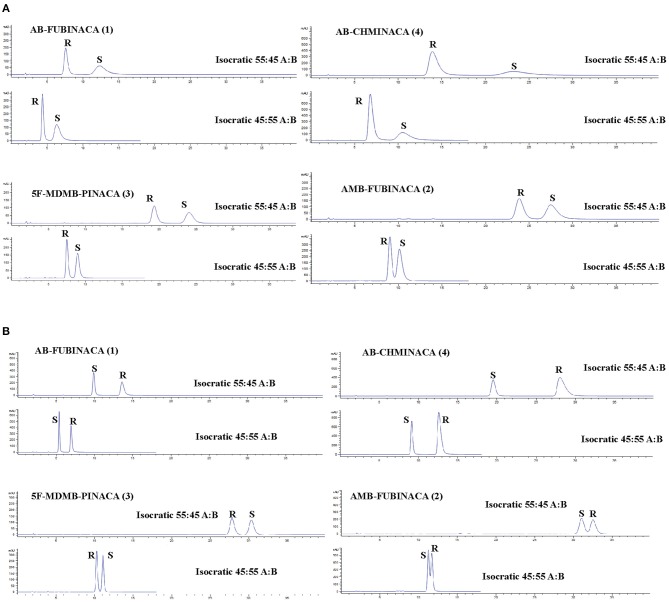
HPLC-PDA chromatograms showing the resolution of indazole-3-carboxamide synthetic cannabinoid receptor agonists on **(A)** a Lux® Amylose-1 column and **(B)** a Lux® i-Cellulose-5 column. Mobile phase components: A-H_2_O, B-Acetonitrile. Compound numbering relates to that provided in Figure 1.

The separation of all four enantiomer pairs on the Amylose-1 column and the i-Cellulose-5 column using either an 45:55 or 55:45 (H_2_O:ACN) isocratic method is shown in Figures 4A,B. Table S1 details the chromatographic parameters [described succinctly by (Chromacademy, 2018)] for both columns using mobile phase compositions of 45:55, 50:50, and 55:45 (H_2_O:ACN) operating in isocratic mode. Although less efficient than the Lux® i-Cellulose-5, the Lux® Amylose-1 column demonstrated greater selectivity and chromatographic performance for SCRA racemates containing terminal methyl esters (AMB-FUBINACA **(2)** and 5F-MDMB-PINACA **(3)**) and with the *(R)*-enantiomer eluting before the *(S)*-enantiomer for all compounds. This is consistent with previously reported chiral separations of similar terminal methyl ester-containing SCRA racemates on amylose-based CSPs (Andernach et al., 2016; Doi et al., 2016, 2018; Weber et al., 2016). In contrast, the Lux® i-Cellulose-5 column demonstrated greater selectivity for SCRA racemates containing a terminal amide moiety (AB-FUBINACA **(1)** and AB-CHMINACA **(4)**). For three of the four compounds the order of elution was reversed on this column, with *(S)*-enantiomers eluting first, except for 5F-MDMB-PINACA **(3)** enantiomers, which retained the same elution order ((*R*) then (*S*)) observed on the Lux® Amylose-1 column. This is the first time a cellulose-based CSP has been reported for the fully resolved separation of SCRA racemates. During the initial screening process (Table 2), the cellulose-based CSPs that contained a chlorinated CSP (e.g., Lux® Cellulose-2 and−4 and Lux® i-Cellulose-5) all showed good selectivity for the AB-FUBINACA **(1)** enantiomers but the non-chlorinated cellulose-based CSP, Lux® Cellulose 1 and 3, were not selective. Doi et al. (2016) previously investigated a CHIRALPAK® OD3 cellulose-based CSP column (cellulose *tris*(3,5-dimethylphenylcarbamate)) for two methyl ester-containing SCRAs, resulting in poor resolution; however, no SCRAs with terminal amide moieties were tested and the CSP selected for testing did not include a chloride moiety. This indicates the importance of the chloride-SCRA terminal amide interaction with this CSP and its role in selectivity. Such knowledge of the separation mechanisms of SCRA enantiomers will aid the choice of CSPs for future studies as new chiral SCRAs emerge onto the illicit drug market.

### Achiral Analysis of Seized Samples

The seized samples described in this study had undergone preliminary qualitative analysis by GC-MS (unpublished data). No samples obtained by MANDRAKE between 2017 and January 2018 were found to contain AB-FUBINACA **(1)**, first detected in Japan in 2012 (Uchiyama et al., 2013) or AB-CHMINACA **(4)**, first detected in 2014 (EMCDDA, 2017c). The production and export of these compounds were controlled in China in January 2015 (UNODC, 2015) and internationally controlled in November 2018 (UNODC, 2018c). AMB-FUBINACA **(2)** and 5F-MDMB-PINACA **(3)** were controlled by China in September 2018^2^ (UNODC, 2018b). Samples were pre-selected to contain either 5F-MDMB-PINACA or AMB-FUBINACA in order to carry out a preliminary study on (*R*)-enantiomer prevalence in seized samples.

**Figure 5:**
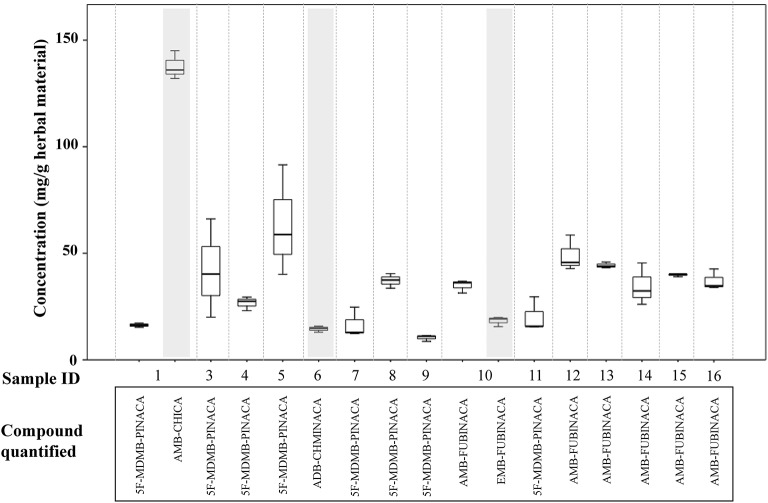
Concentrations of synthetic cannabinoid receptor agonists (SCRAs) seized in Manchester, UK. Shaded areas denote samples where a semi-quantitative result has been calculated for a compound based on the detector response for AMB-FUBINACA.

The results of the qualitative and quantitative analysis of the selected seized samples are described in Table 3 and in Figure 5. In order to estimate herbal material heterogeneity, samples were not homogenized prior to extraction. There was variability in the concentrations detected in the replicate samples, reflecting the inherent heterogeneity of such herbal materials, with 2 to 3-fold differences in concentration being detected in single samples. This is likely to add to variability in the effects on the consumer if the materials are subsampled for use by consumers at different times and so therefore, from a harm reduction and risk assessment context it is helpful to analyse and report such samples in this way.

**Table 3:** Synthetic cannabinoid receptor agonists (SCRAs) in seized samples from Manchester, UK.

**Sample Information**		**GC-MS**		**UPLC**^**®**^**-QToF-MS/MS**		
**Sample ID**	**Date seized**	**SCRA detected (reverse match factor to library hit)**	**Concentration mean (range) mg/g, *n* = 3**	**Molecular Accurate Mass [M+H]^**+**^ (mass error, ppm)**	**[M+H] and characteristic ions**	**Chiral Profile S:R (%)**
1	21/03/17	AMB-CHMICA (901) 5F-MDMB-PINACA (931)	137 (132-145)^*^ 16.2 (15.2-17.2)	371.2336 (1.1) 378.2188 (-2.6)	240.1375, 144.0439, 97.0998 233.1096, 318.1975, 213.1026	– 93.6: 6.4
2	31/03/17	5F-MDMB-PINACA (931)	Insufficient sample	378.2193 (−1.3)	233.1091, 318.1976, 213.1018	99.8: 0.2
3	11/04/17	5F-MDMB-PINACA (931)	42.1 (20.0-66.1)	378.2199 (0.3)	233.0949, 318.1757, 213.0904	99.3: 0.7
4	11/04/17	5F-MDMB-PINACA (931)	26.6 (23.0-29.4)	378.2202 (1.1)	233.0953, 318.1761, 213.0908	99.3: 0.7
5	31/07/17	5F-MDMB-PINACA (931)	63.5 (40.1-91.5)	378.2198 (0.0)	233.0950, 318.1759, 213.0950	99.3: 0.7
6	31/07/17	ADB-CHNACA (919) 5F-ADB-PINACA (832) 5F-MDMB-PINACA (936)	14.4 (12.9–15.8)^*^ < 1.00 < 1.00	371.2446 (1.1) 363.2188 (−0.6) 378.2183 (−4.0)^$^	326.2221, 241.1330, 354.2169 318.1967, 233.10746.1891 318.1949, 233.1043, 346.1919	– – –
7	31/07/17	5F-MDMB-PINACA (931)	16.6 (12.3-24.7)	378.2211 (3.4)	233.0950, 318.1756, 213.0903	99.3: 0.7
8	22/10/17	5F-MDMB-PINACA (931)	37.1 (33.6-40.4)	378.2207 (2.4)	233.0952, 318.1761, 213.0905	99.3: 0.7
9	22/10/17	5F-MDMB-PINACA (931)	14.4 (12.9-15.8)	378.2204 (1.6)	233.0940, 318.1741, 213.0895	99.3: 0.7
10	15/01/18	AMB-FUBINACA (940) EMB-FUBINACA (933)	52.3 (47.5-54.8) 27.4 (23.6-30.0)^*^	384.1723 (1.3) 398.1870 (−1.0)	253.0782, 324,1508, 324.1508 253.0765, 109.0432, 324.1492	96.8: 3.2 -
11	15/01/18	5F-MDMB-PINACA (933)	20.2 (15.5–29.5)	378.2192 (-1.6)	233.1093, 318.1976, 213.1020	99.1: 0.90
12	05/01/18	AMB-FUBINACA (913)	49.0 (42.8–58.5)	384.1718 (0.0)	253.0789, 109.048, 324.1521	98.2: 1.80
13	05/01/18	AMB-FUBINACA (913)	44.3 (43.2–45.9)	384.1720 (0.5)	253.0793, 109.0479, 324.1526	98.2: 1.80
14	05/01/18	AMB-FUBINACA (913)	34.6 (26.0–45.4)	384.1720 (0.5)	253.0788, 109.0467, 324.1528	98.2: 1.79
15	05/01/18	AMB-FUBINACA (913)	39.9 (39.1–40.4)	384.1719 (0.3)	253.0788, 109.0474, 324.1525	98.2: 1.80
16	05/01/18	AMB-FUBINACA (913)	37.1 (22.8–28.7)	384.1720 (0.5)	253.0787, 109.0466, 324.1523	98.2: 1.80

* Semi-quantitative data calculated using the GC-MS detector response for AMB-FUBINACA;

$ *Very low signal giving rise to a poor quality MS/MS spectrum*.

5F-MDMB-PINACA **(3)** was detected by GC-MS as the only SCRA present in six samples and as part of a mixture in two samples. The concentrations of 5F-MDMB-PINACA in the samples ranged from < 1.0 to 91.5 mg/g herbal material (< 0.1 to 9.15% w/w herbal material). It was a minor SCRA component in one sample, where it was detected with AMB-CHMICA **(16)** (methyl 2-(1-(cyclohexylmethyl)-1*H*-indole-3-carboxamido)-3-methylbut-anoate), also known as MMB-CHMICA. AMB-CHMICA has previously been determined to be six times less potent than 5F-MDMB-PINACA (Banister et al., 2016). 5F-MDMB-PINACA **(3)** was also present as a trace component (< 1 mg/g, < 0.1% w/w herbal material) of a mixture with ADB-CHMINACA **(17)** (*N*-(1-amino-3,3-dimethyl-1-oxobutan-2-yl)-1-(cyclohexylmethyl) indazole-3-carboxamide), also known as MAB-CHMICA, and traces of tentatively identified 5F-ADB-PINACA isomer 2 **(18)** (*N*-(1-amino-3,3-dimethyl-1-oxo-2-butan-2-yl)-1-(5-fluoro-pentyl)-1*H*-indazole-3-carboxamide). Interestingly, ADB-CHMINACA **(17)** has been demonstrated to be equipotent with 5F-MDMB-PINACA **(3)** (Banister et al., 2015a).

AMB-FUBINACA **(2)** was detected in six samples, in five as the only SCRA present and in one sample mixed with the closely structurally-related SCRA, EMB-FUBINACA **(10)** (ethyl 2-(1-(4-fluorobenzyl)-1H-indazole-3-carboxamido)-3-methylbutanoate). The concentrations of AMB-FUBINACA **(2)** ranged from 15.5 to 58.5 mg/g herbal material (1.55–5.85% w/w herbal material). AMB-FUBINACA **(2)** was previously detected in a product known as “AK-47 24 Karat Gold,” which was implicated in an acute toxicity event in New York in 2016 (Adams et al., 2017). Replicate analysis of a single packet of the material in this case gave a result of 16.0 +/– 3.9 mg/g (1.6% w/w herbal material). Five of the seized samples reported in this study were seized on the same day in the same area as each other and all contain relatively similar concentrations of AMB-FUBINACA (range 22.8–58.5 mg/g herbal material). Semi-quantitative estimates have been provided for the concentrations of AMB-CHMICA **(16)**, ADB-CHMINACA **(17)** and EMB-FUBINACA **(10)** in the samples. As such, the data should be treated as indicative only.

As well as the GC-MS data, the molecular mass and fragmentation patterns of these compounds were confirmed by HRMS analysis (see Table 3), with reference being made to previously published comparative data on the same compounds (Carlier et al., 2017a,b; Liu et al., 2017; Mardal et al., 2018). All mass assignments agreed within 4.1 ppm with calculated theoretical monoisotopic masses (Table 3).

### Chiral Analysis of Seized Samples

**Figure 6:**
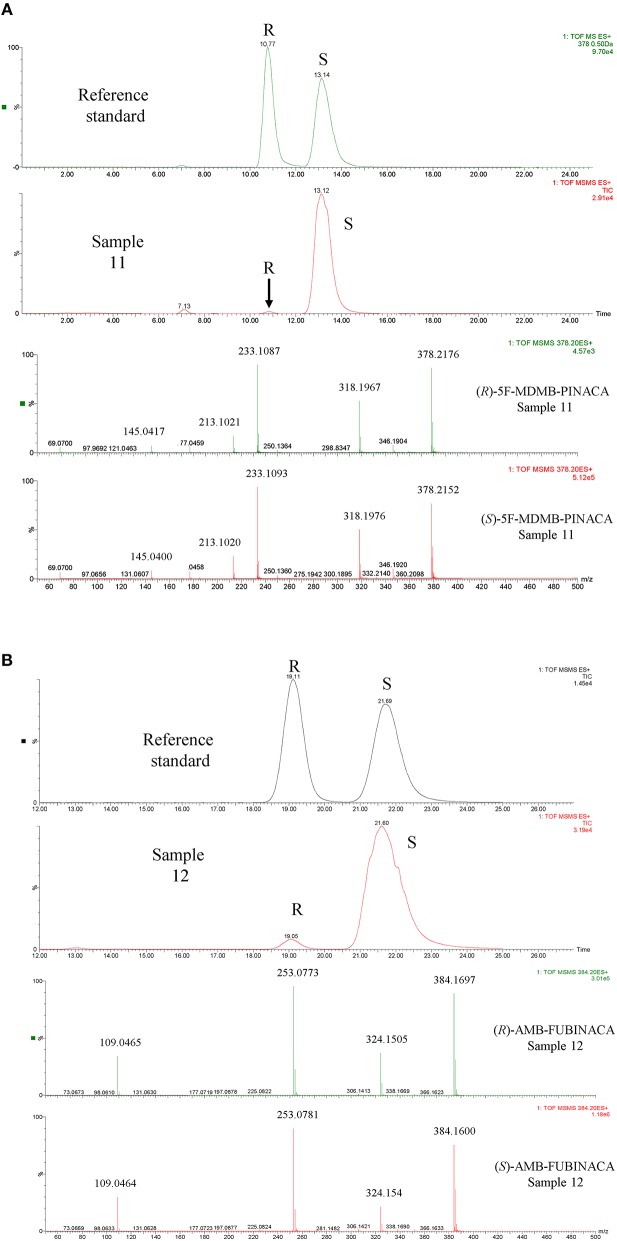
Examples of the identification of SCRA enantiomers in seized samples by Chiral UPLC®-QToF-MS/MS **(A)** sample 11 containing 5F-MDMB-PINACA and **(B)** sample 12 containing AMB-FUBINACA. The most important accurate mass data has been enlarged in the spectra provided for ease of viewing.

Enantiomer ratios were determined using the relative peak areas of the chromatographically resolved enantiomers rather than a calibration curve due to the very large difference in concentrations of each enantiomer detected in the samples (Table 3). Due to its wide linear range, the PDA detector response at 210 nm was used for this purpose. It was found that the calculation of the enantiomer ratio using the HRMS response led to an over-estimation of the contribution of the minor (*R*)-enantiomer. This was confirmed by analysis of 10-, 100-, and 1,000-fold dilutions of the original extract. The HRMS detector was used for confirmatory purposes and ToF-MS, MS^e^ and MS/MS data provided confirmation of enantiomer identification in addition to retention time matching to a racemic reference standard (see Figure 6 for examples of AMB-FUBINACA **(2)** and 5F-MDMB-PINACA **(3)** enantiomer identification). The ratio calculated using the PDA detector remained constant over the dilution range and the ratio calculated using the HRMS detector decreased with increasing sample dilution. The chiral profiles of AMB-FUBINACA **(2)** and 5F-MDMA-PINACA **(3)** and in 16 seized herbal materials are shown in Table 3. The limited chiral profiling studies carried out to date on seized SCRAs have reported the expected and overwhelming prevalence of the (*S)*-enantiomers in recovered samples (Andernach et al., 2016; Doi et al., 2016; Weber et al., 2016). Some variation in the enantiopurity of precursors may occur as a result of variation in the supply chain and could therefore be utilized in SCRA batch profiling if such variation were reproducible within a production batch or supplier. L-*tert*-leucine, the precursor of 5F-MDMB-PINACA (and other leucine-derived SCRAs as well as licit pharmaceuticals such as antiretroviral protease inhibitors e.g., Atazanavir, Boceprevir, and Telaprevir) is widely available and is produced efficiently on an industrial scale with a high degree of enantiopurity (>99%) (Xue et al., 2018).

In this study, as expected from previous studies and as a result of the lower cost and the relative availability of the relevant precursors, the more potent (*S*)-enantiomer of 5F-MDMB-PINACA **(3)** was the major enantiomer, present at >93% in all samples analyzed and in the majority at >99%. (*S*)-AMB-FUBINACA **(2)** was present at 96.8–98.2 % in the small sample set examined. An (almost) identical ratio was obtained for the 5 samples seized on the same day, with this ratio being distinct from the other sample, indicating that chiral profiling may be a useful additional tool in distinguishing batches of valine methyl ester-derived SCRAs at a synthetic source level even though the absolute size of the variation in chiral profiles is small.

### Estimated Intrinsic Potency of Seized Samples

We propose that the intrinsic potency of one SCRA containing sample relative to another can be estimated using the CB_1_ potency of each compound detected, relative to the control sample used in the bioassay (in this case JWH-018) and the concentration of each SCRA, with a correction factor applied for enantiopurity where such information is available. The calculation of such an estimated intrinsic potency provides a simple methodology for comparing the relative potential for harm of different sample batches containing a number of SCRA compounds at different concentrations for risk evaluation, harm reduction and/or intelligence purposes. This approach is a method of comparing samples before they are consumed and does not directly indicate the physiological/toxicological effects that would be experienced by a user following exposure. The estimated intrinsic potency calculation does not currently take into account the potential effect of differences in CB_1_ efficacy between the SCRAs. Within this study, the range of E_max_ values calculated for the different (S)-enantiomers was relatively small (267–331%), therefore the inclusion of the Emax values in the estimated intrinsic potency calculation would have a limited effect on the differentiation of seized samples. It should be noted, though, that comparing E_max_ values (as a measure of efficacy) from different studies can result in different outcomes. Sachdev et al. (2018) have developed an interesting approach to compare the efficacy of SCRAs (also taking into account the potential “assay ceiling” issue), which could also be used in this method to assess the potency of seized samples. User response will be affected by a number of additional factors, including user tolerance, the presence of other co-consumed substances and the influence of the mode of use of the drug e.g., smoking vs. vaping vs. ingestion. During smoking, active and inactive pyrolysis products may be produced which would then be inhaled (Kevin et al., 2019). The physiological response of a user to a sample could also be affected by the rate of metabolism of the parent SCRA(s) following exposure, the formation of pharmacologically active metabolites (Cannaert et al., 2016; Gamage et al., 2019) and the relative ability of both parent and metabolites to pass the blood-brain barrier to exert their CB_1_ mediated psychoactive effects.

Keeping in mind the above-mentioned limitations, the estimated intrinsic potency of the SCRA-containing sample can be calculated as described in Equations (1) and (2).

When the relative proportions of the enantiomers have not been determined:

(1) $$\begin{array}{r} Estimated\;Intrinsic\;Potency=\;\frac{\left[\left(\frac{{\text{CB}1\;EC}_{50,\;JWH-018}}{{\text{CB}1\;EC}_{50,\;SCRA}}\right)\text{x }Conc_{SCRA}\right]}{100} \end{array}$$

When the relative proportions of the enantiomers have been determined they can be used to adjust the estimated intrinsic potency value:

(2) $$\begin{array}{l} Estimated\;Intrinsic\;Potency\\ =\frac{\left[\left(\frac{{\text{CB}1\;EC}_{50,\;JWH-018}}{{\text{CB}1\;EC}_{50,\;SCRA}}\right)\text{x }P\left(S\right)\right]+\left[\left(\frac{{\text{CB}1\;EC}_{50,\;JWH-018}}{{\text{CB}1\;EC}_{50,\;SCRA}}\right)\text{x }P\left(R\right)\;\right]*Conc_{SCRA}}{100} \end{array}$$

Where,

EC_50_ values are expressed in nM, Conc_SCRA_ is the concentration (mg/g) of a particular SCRA detected in the sample, P(*S*) is the proportion of the (*S*)-enantiomer present in the sample e.g., 93.6% = 0.936, P(*R*) is the proportion of the (*R*)-enantiomer present in the sample e.g., 6.4% = 0.064.

When multiple SCRAs are present in a single sample the term on the top line is simply repeated for each SCRA detected and added together. A combination of the two equations can be used when chiral data is available for some SCRAs but not for others.

**Figure 7:**
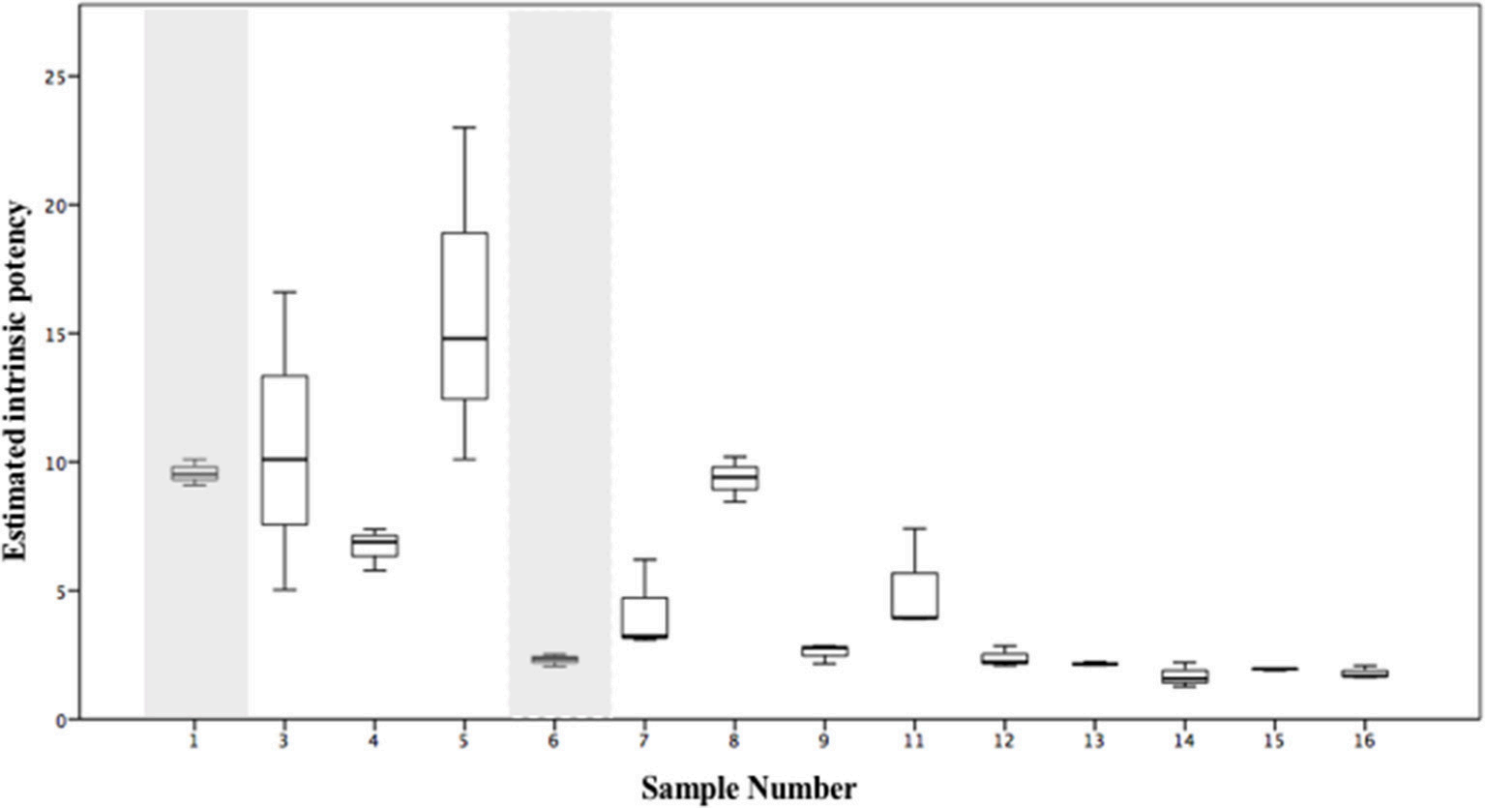
Estimated intrinsic potency values, calculated using equation 1 and/or equation 2, from replicate analyses (*n* = 3) of alleged “spice” samples seized in Manchester, UK. Shaded areas identify samples for which estimated intrinsic potency values have been calculated. Intrinsic potency values for sample 2 (see Table 3) have not been calculated due to insufficient sample for quantitative analysis. Sample 10 (see Table 3), in which both AMB-FUBINACA and EMB-FUBINACA were detected has also been omitted as no comparative data for potency (EC_50_) is currently available for EMB-FUBINACA.

The estimated intrinsic potency values have been calculated for the samples seized in this study which contained either 5F-MDMB-PINACA or AMB-FUBINACA (Figure 7) and the underlying data and calculations are provided in Tables S2, S3a,b. To illustrate how such an approach could be used when multiple SCRAs are present in samples, an estimated intrinsic potency value has been calculated for compounds which have been quantified on a semi-quantitative basis and whose potency (EC_50_) has not been determined directly in this study. For example, 5F-MDMB-PINACA and AMB-CHMICA were both detected in sample 1. As no EC_50_ data is available for AMB-CHMICA from this study, the EC_50_ for 5F-MDMB-PINACA was used and a correction factor applied. This correction factor was derived from data reported by Banister et al. (2016) who have previously determined the EC_50_ values for both compounds: correction factor = (EC_50_, _5F−MDMB−PINACA_ / EC_50_, _AMB−CHMICA_) = (0.59/3.59) = 0.164. ADB-CHMINACA was detected in sample 6 and the EC_50_ value relative to JWH-018 was calculated using the data reported previously by Cannaert et al. (2017). As no relative potency data is available for EMB-FUBINACA, either to another compound in this study or to a JWH-018 control sample run using the same assay, no intrinsic potency values were calculated for sample 10 which contained both AMB-FUBINACA and EMB-FUBINACA. The estimated intrinsic potency varies even within this small sample with its limited variability of SCRA compounds (Figure 7 and in Table S2). The samples with the lowest estimated intrinsic potency in this study are up to 10 times less potent than the most potent samples. Coupled with the heterogeneous nature of the herbal samples this will inevitably lead to unpredictable effects experienced by the user(s). The visual characteristics of SCRA infused materials give no indication of the SCRAs present, their concentration or their relative potency.

We propose that bioassay data on pure compounds (and where available, data on the prevalence of both the (*R*)- and (*S*)-enantiomers), is compiled for existing and emerging SCRAs to allow the calculation of estimated intrinsic potency values as a standard practice for evaluating and comparing the potential harms of SCRA containing materials. If present, signaling bias caused by different SCRAs may affect the accuracy of estimated intrinsic potency and so this also warrants further study. Alternatively, the bioassay could be used as a standalone assessment technique to determine a samples EC_50_ without the necessity for identification of the SCRA components or their concentrations. Either way, the calculation of an intrinsic potency value allows independent samples, containing different SCRAs with inherently different potencies (as expressed by the EC_50_) at different concentrations to be compared directly with each other. This may be a useful tool in expressing differences in the relative potential harms of seized samples.

## Conclusions

In this study, we have described the investigation of the effect of chirality on the potency and, for the first time, efficacy, of four indazole-3-carboxamide SCRAs. All (*S*)-enantiomers were more potent than their respective (*R*)-enantiomers at both CB_1_ and CB_2_ although the scale of the difference varied greatly between compounds. (*S*)-enantiomers had up to three times greater efficacy at CB_1_ but had similar or lower efficacy at CB_2_ than their respective (*R*)-counterparts. To assess the prevalence and variability of (*R*)-enantiomers of SCRAs in seized samples, an analytical method was developed for the separation of SCRA enantiomers. With an increased knowledge of chiral column retention mechanisms and selectivity, we have recommended two column types, depending on the structural features of the SCRAs studied. With such knowledge, methods can be selected and adapted as new chiral SCRAs enter the market. We have successfully applied the method to a small number (n = 16) of seized samples in a preliminary study where all SCRAs present have been identified and quantified where possible. It is recommended that amylose-based CSPs are used for the analysis of SCRAs with terminal methyl esters (e.g., 5F-MDMB-PINACA and AMB-FUBINACA in this study) and that cellulose-based chlorinated CSPs are used for those compounds with terminal amides (e.g., AB-FUBINACA and AB-CHMINACA in this study). A considerably larger set of samples will be analyzed in future to determine within and between batch variation of chiral profiles and their potential discriminatory power in case samples.

We have proposed a methodology for calculating the estimated intrinsic potency of a drug sample as presented to a user, prior to use. Due to the utilization of different signaling pathways in different *in vitro* bioassays for the evaluation of potency and efficacy of SCRAs at CB_1_ and CB_2_ receptors, we recommend further investigation of the possible impact of SCRA signaling bias on the estimated intrinsic potency approach introduced in this study. A direct comparison of the *in vitro* response of the bioassay to both G-protein and β-arrestin mediated pathways in the same assay or between independent assay systems (e.g., the assay used in this study vs. FLIPR membrane potential assay *vs*. GTPγS binding assay vs. cAMP accumulation assays) is recommended. At present the estimated intrinsic potency calculated for a sample does not take into account the efficacy of the SCRAs present and this warrants further investigation.

Further experiments could investigate the effect of chirality on SCRA potency and efficacy using the following compounds and their enantiomers: (*S*)- and (*R*)-MDMB-FUBINACA vs. (*S*) and (*R*)-AMB-FUBINACA and (*S*)- and (*R*)-5F-MDMB-PINACA vs. (*S*)- and (*R*)-5F-AMB-PINACA. Such a study would further enhance our understanding of the mechanism of SCRA-CB_1_ receptor interactions.

## Data Availability

Datasets generated for this study are included in the manuscript and the Supplementary Files. Any additional raw data supporting the conclusions of this work will be made available by the authors, without undue reservation, to any qualified researcher.

## Author Contributions

CM, OS, and CS participated in research study design. LA, CN, ACa, ACo, AH, LV, and CM carried out laboratory analysis. LA, ACa, CN and CM performed data analysis. LA, ACa, NN, OS, CS, and CM wrote or contributed to the writing of the manuscript.

### Conflict of Interest Statement

The authors declare that the research was conducted in the absence of any commercial or financial relationships that could be construed as a potential conflict of interest.

## Footnotes

^1^https://www.thepaper.cn/newsDetail_forward_2389954 (accessed February 2, 2019)

^2^https://www.thepaper.cn/newsDetail_forward_2389954 (accessed February 2, 2019).

## References

[B1] Adamowicz P. (2016). Fatal intoxication with synthetic cannabinoid MDMB-CHMICA. For. Sci. Int. 261, e5–10. 10.1016/j.forsciint.2016.02.024 26934903

[B2] Adams A. J. Banister S. D. Irizarry L. Trecki J. Schwartz M. Gerona R. (2017). “Zombie” outbreak caused by the synthetic cannabinoid AMB-FUBINACA in New York. N. Engl. J. Med. 376, 235–242. 10.1056/NEJMoa1610300 27973993

[B3] Adebar Project (2018). Analytical Report: 5F-AB-P7AICA, N-(1-Amino-3-methyl-1-oxobutan-2-yl)-1-(5-fluoropentyl)-1H-pyrrolo- [2,3-b]pyridine-3-carboxamide. Available online at: https://www.policija.si/apps/nfl_response_web/0_Analytical_Reports_final/5F-AB-P7AICA-ID-ADB-18_076_report.pdf (accessed February 2, 2019).

[B4] Andernach L. Pusch S. Weber C. Schollmeyer D. Münster-Müller S. Pütz M. (2016). Absolute configuration of the synthetic cannabinoid MDMB-CHMICA with its chemical characteristics in illegal products. For. Toxicol. 34, 344–352. 10.1007/s11419-016-0321-1

[B5] Angerer V. Franz F. Moosmann B. Bisel P. Auwärter V. (2019). 5F-Cumyl-PINACA in ‘e-liquids' for electronic cigarettes: comprehensive characterization of a new type of synthetic cannabinoid in a trendy product including investigations on the in vitro and in vivo phase I metabolism of 5F-Cumyl-PINACA and its non-fluorinated analog Cumyl-PINACA. For. Toxicol. 37, 186–196. 10.1007/s11419-018-0451-8 30636986 PMC6315005

[B6] Angerer V. Jacobi S. Franz F. Auwarter V. Pietsch J. (2017). Three fatalities associated with the synthetic cannabinoids 5F-ADB, 5F-PB-22, and AB-CHMINACA. For. Sci Int. 281, e9–e15. 10.1016/j.forsciint.2017.10.042 29133010

[B7] Banister S. D. Adams A. Kevin R. C. Macdonald C. Glass M. Boyd R.. (2019b). Synthesis and pharmacology of new psychoactive substance 5F-CUMYL-P7AICA, a scaffold- hopping analog of synthetic cannabinoid receptor agonists 5F-CUMYL-PICA and 5F-CUMYL-PINACA. Drug Test. Anal. 11, 279–291. 10.1002/dta.2491 30151911

[B8] Banister S. D. Connor M. (2018a). The chemistry and pharmacology of synthetic cannabinoid receptor agonist new psychoactive substances: origins. Handb. Exp. Pharmacol. 252, 165–190. 10.1007/164_2018_143 29980914

[B9] Banister S. D. Connor M. (2018b). The chemistry and pharmacology of synthetic cannabinoid receptor agonist new psychoactive substances: evolution. Handb. Exp. Pharmacol. 252, 191–226. 10.1007/164_2018_144 30105473

[B10] Banister S. D. Kevin R. C. Martin L. Adams A. Macdonald C. Manning J. J.. (2019a). The chemistry and pharmacology of putative synthetic cannabinoid receptor agonist (SCRA) new psychoactive substances (NPS) 5F-PY-PICA, 5F-PY-PINACA, and their analogues. Drug Test Anal. [Epub ahead of print]. 10.1002/dta.2583 30838752

[B11] Banister S. D. Longworth M. Kevin R. Sachdev S. Santiago M. Stuart J.. (2016). Pharmacology of Valinate and tert-Leucinate Synthetic Cannabinoids 5F-AMBICA, 5F-AMB, 5F-ADB, AMB-FUBINACA, MDMB-FUBINACA, MDMB-CHMICA, and Their Analogues. ACS Chem. Neuro. 7, 1241–1254. 10.1021/acschemneuro.6b00137 27421060

[B12] Banister S. D. Moir M. Stuart J. Kevin R. C. Wood K. E. Longworth M.. (2015a). Pharmacology of indole and indazole synthetic cannabinoid designer drugs AB-FUBINACA, ADB-FUBINACA, AB-PINACA, ADB-PINACA, 5F-AB-PINACA, 5F-ADB-PINACA, ADBICA, and 5F-ADBICA. ACS Chem. Neuro. 6, 1546–1559. 10.1021/acschemneuro.5b00112 26134475

[B13] Banister S. D. Stuart J. Kevin R. C. Edington A. Longworth M. Wilkinson S. M. (2015b). Effects of bioisosteric fluorine in synthetic cannabinoid designer drugs JWH-018, AM-2201, UR-144, XLR11, PB-22, 5F-PB-22, APICA, and STS-135. ACS Chem. Neurosci. 6, 1445–1458. 10.1021/acschemneuro.5b00107 25921407

[B14] Banister S. D. Wilkinson S. M. Longworth M. Stuart J. Apetz N. English K.. (2013). The synthesis and pharmacological evaluation of adamantane-derived indoles: cannabimimetic drugs of abuse. ACS Chem. Neurosci. 4, 1081–1092. 10.1021/cn400035r 23551277 PMC3715837

[B15] Berthod A. (2010). “Chiral recognition mechanisms in enantiomers separations: a general view,” in Chiral Recognition in Separation Methods, ed Berthod A. (Berlin; Heidelberg: Springer). 10.1007/978-3-642-12445-7_1

[B16] Buchler I. P. Hayes M. J. Hedge S. G. Hockerman S. L. Jones D. E. Kortum S. W. (2009). Indazole Derivatives as CB1 Receptor Modulators and Their Preparation and Use in the Treatment of CB1-Mediated Diseases. Patent WO 2009/106982, New York, NY: Pfizer Inc.

[B17] Cannaert A. Franz F. Auwärter V. Stove C. P. (2017). Activity-based detection of consumption of synthetic cannabinoids in authentic urine samples using a stable cannabinoid reporter system. Anal. Chem. 89, 9527–9536. 10.1021/acs.analchem.7b02552 28771321

[B18] Cannaert A. Storme J. Franz F. Auwärter V. Stove C. P. (2016). Detection and activity profiling of synthetic cannabinoids and their metabolites with a newly developed bioassay. Anal. Chem. 88, 11476–11485. 10.1021/acs.analchem.6b02600 27779402

[B19] Cannaert A. Storme J. Hess C. Auwärter V. Wille S. M. R. Stove C. P. (2018). Activity-based detection of cannabinoids in serum and plasma samples. Clin. Chem. 64, 918-926. 10.1373/clinchem.2017.285361 29559524

[B20] Cannaert A. Vandeputte M. Hudson S. Wood D. M. Dargan P. I. Stove C. P. (2019a). Validation of activity-based screening for synthetic cannabinoid receptor agonists in a large set of serum samples. Clin. Chem. 65, 347–349. 10.1373/clinchem.2018.296905 30504261

[B21] Cannaert A. Vandeputte M. Wille S. M. R. Stove C. P. (2019b). Activity-based reporter assays for the screening of abused substances in biological matrices. Crit. Rev. Toxicol. 1–15. [Epub ahead of print]. 10.1080/10408444.2019.1576588 30919714

[B22] Carlier J. Diao X. Scheidweiler K. B. Huestis M. A. (2017a). Distinguishing intake of new synthetic cannabinoids ADB-PINACA and 5F-ADB-PINACA with human hepatocyte metabolites and high resolution mass spectrometry. Clin. Chem. 63, 1008–1021. 10.1373/clinchem.2016.267575 28302730

[B23] Carlier J. Diao X. Sempio C. Huestis M. A. (2017b). Identification of new synthetic cannabinoid ADB-CHMINACA (MAB-CHMINACA) metabolites in human hepatocytes. AAPS J. 19, 568–577. 10.1208/s12248-016-0037-5 28070717

[B24] Centre for Forensic Science Education (2018). Trend Report: Q3 2018. Synthetic Cannabinoids in the United States. Available online at: https://www.forensicscienceeducation.org/wp-content/uploads/2019/02/Synthetic-Cannabinoid-Trend-Report_Detailed_2018-Q3_revised012419.pdf (accessed February 2, 2019).

[B25] Centre for Forensic Science Education (2019). New Synthetic Cannabinoid: 4F-MDMB-BINACA. Available online at: https://www.forensicscienceeducation.org/wp-content/uploads/2019/01/4F-MDMB-BINACA_011118_NMSLabs_Report.pdf (accessed February 2, 2019).

[B26] Chromacademy (2018). The Theory of HPLC Chromatographic Parameters. Available online at: https://www.chromacademy.com/lms/sco2/Theory_Of_HPLC_Chromatographic_Parameters.pdf (accessed November 14, 2018).

[B27] DEA (2018a). Emerging Threat Report. 3rd Quarter 2018. Available online at: https://ndews.umd.edu/sites/ndews.umd.edu/files/dea-emerging-threat-report-2018-quarter-3.pdf (accessed February 2, 2019).

[B28] DEA (2018b). Emerging Threat Report. Mid-year 2018. Available online at: https://ndews.umd.edu/sites/ndews.umd.edu/files/dea-emerging-threat-report-2018-mid-year.pdf (accessed February 2, 2019).

[B29] Doi T. Asada A. Takeda A. Tagami T. Katagi M. Kamata H.. (2016). Enantioseparation of the carboxamide-type synthetic cannabinoids N-(1-amino-3-methyl-1-oxobutan-2-yl)-1-(5-fluoropentyl)-1H-indazole-3-carboxamide and methyl [1-(5-fluoropentyl)-1H-indazole-3-carbonyl]-valinate in illicit herbal products. J. Chromatogr. A. 1473, 83–89. 10.1016/j.chroma.2016.10.049 27773389

[B30] Doi T. Asada A. Takeda A. Tagami T. Katagi M. Kamata H.. (2018). Evaluation of carboxamide-type synthetic cannabinoids as CB1/CB2 receptor agonists: difference between the enantiomers. For. Toxicol. 36, 51–60. 10.1007/s11419-017-0378-5 29367862 PMC5754384

[B31] EMCDDA (2017a). Synthetic Cannabinoids in Europe. Perspectives on Drugs. Lisbon: EMCDDA 10.2810/32306

[B32] EMCDDA (2017b). Joint Report on a New Psychoactive Substance: N-(1-amino-3,3-dimethyl-1-oxobutan2-yl)-1-(cyclohexylmethyl)-1H-indazole-3-carboxamide (ADB-CHMINACA). In Accordance With Article 5 of Council Decision 2005/387/JHA on the Information Exchange, Risk Assessment and Control of New Psychoactive Substances. Lisbon: EMCDDA-Europol 10.2810/41095

[B33] EMCDDA (2017c). Joint Report on a New Psychoactive Substance: N-(1-amino-3-methyl-1-oxobutan-2-yl)-1-(cyclohexylmethyl)-1H-indazole-3-carboxamide(AB-CHMINACA) In Accordance With Article 5 of Council Decision 2005/387/JHA on the Information Exchange, Risk Assessment and Control of New Psychoactive Substances. Lisbon: EMCDDA-Europol 10.2810/210307

[B34] EMCDDA (2017d). Joint Report on a New Psychoactive Substance: methyl 2-{[1-(5-fluoropentyl)-1hindazole-3-carbonyl]amino}-3,3-dimethylbutanoate (5F-MDMB-PINACA; 5F-ADB). In Accordance With Article 5 of Council Decision 2005/387/JHA on the Information Exchange, Risk Assessment and Control of New Psychoactive Substances. Lisbon: EMCDDA-Europol.

[B35] EMCDDA (2018a). European Drug Report 2018. European Drug Report 2018: Trends and Developments. Luxembourg: EMCDDA 10.2810/800331

[B36] EMCDDA (2018b). Fentanils and Synthetic Cannabinoids: Driving Greater Complexity Into the Drug Situation. Lisbon: EMCDDA.

[B37] EMCDDA (2018c). New Psychoactive Substances in Prison. Lisbon: EMCDDA.

[B38] EMCDDA (2019). Formal Notification of N-(1-amino-1-oxo-3-phenylpropan-2-yl)-1-butyl-1H-indazole-3-carboxamide (APP-BINACA) by the United Kingdom as a New Psychoactive Substance Under the Terms of Regulation (EU) 2017/2101 (Lisbon).

[B39] Ford L. T. Berg J. D. (2018). Analytical evidence to show letters impregnated with novel psychoactive substances are a means of getting drugs to inmates within the UK prison service. Ann. Clin. Biochem. 55, 673–678. 10.1177/0004563218767462 29534614

[B40] Frinculescu A. Lyall C. L. Ramsey J. Miserez B. (2017). Variation in commercial smoking mixtures containing 22 Fentanils and synthetic cannabinoids: driving greater complexity into the drug situation third-generation synthetic cannabinoids. Drug Test. Anal. 9, 327–333, 10.1002/dta.1975 27161591

[B41] Funada M. Takebayashi-ohsawa M. (2018). Synthetic cannabinoid AM2201 induces seizures: Involvement of cannabinoid CB1 receptors and glutamatergic transmission. Toxicol. Appl. Pharmacol. 338, 1–8. 10.1016/j.taap.2017.10.007 29042214

[B42] Gamage T. F. Farquhar C. E. McKinnie R. J. Kevin R. C. McGregor I. S. Trudell M. L.. (2019). Synthetic cannabinoid hydroxypentyl metabolites retain efficacy at human cannabinoid receptors. J. Pharmacol. Exp. Ther. Mar. 368, 414–422. 10.1124/jpet.118.254425. 30552295 PMC6374541

[B43] Gieron J. Adamowicz P. (2016). Fatal poisoning with the synthetic cannabinoid AB-CHMINACA and ethyl alcohol - a case study and literature review. Probl. Forensic Sci. 106, 482–495.

[B44] Han S. Thatte J. Buzard D. J. Jones R. M. (2013). Therapeutic utility of cannabinoid receptor type 2 (CB2) selective agonists. J. Med. Chem. 56, 8224–8256. 10.1021/jm4005626 23865723

[B45] Ho J. H. Stahl E. L. Schmid C. L. Scarry S. M. Aubé J. Bohn L. M. (2018). G protein signaling-biased agonism at the κ-opioid receptor is maintained in striatal neurons. Sci. Signal. 11:eaar4309. 10.1126/scisignal.aar4309 30087177 PMC6373773

[B46] Hua T. Vemuri K. Pu M. Qu L. Han G. W. Wu Y.. (2016). Crystal structure of the human cannabinoid receptor CB1. Cell 167, 750–762. 10.1016/j.cell.2016.10.004 27768894 PMC5322940

[B47] Huffman J. W. (2009). “Cannabimimetic indoles, pyrroles, and indenes: structure-activity relationships and receptor interactions,” in The Cannabinoid Receptors, ed. Reggio P. H. (New York, NY, Humana Press), 49–94. 10.1007/978-1-59745-503-9_3

[B48] Huffman J. W. Padgett L. W. (2005). Recent development in medicinal chemistry of cannabimimetic indoles, pyrroles and indenes. Curr. Med. Chem. 12, 1395–1411. 10.2174/0929867054020864 15974991

[B49] Huffman J. W. Zengin G. Wu M.-J. Lu J. Hynd G. Bushell K. (2005). Structure–activity relationships for 1-alkyl-3-(1-naphthoyl)indoles at the cannabinoid CB1 and CB2 receptors: steric and electronic effects of naphthoyl substituents. New highly selective CB2 receptor agonists. Bioorg. Med. Chem. 13, 89–112. 10.1016/j.bmc.2004.09.050 15582455

[B50] Ibsen M. S. Connor M. Glass M. (2017). Cannabinoid CB1 and CB2 receptor signaling and bias. Cann. Cannabi. Res. 2, 48–60. 10.1089/can.2016.0037 28861504 PMC5436336

[B51] Kadkhodaei K. Forcher L. Schmid M. G. (2018). Separation of enantiomers of new psychoactive substances by high-performance liquid chromatography. J. Sep. Sci. 41, 1274–1286. 10.1002/jssc.201701239 29280291

[B52] Kevin R. C. Kovach A. L. Lefever T. W. Gamage T. F. Wiley J. L. Mcgregor I. S.. (2019). Toxic by design? Formation of thermal degradants and cyanide from carboxamide-type synthetic cannabinoids CUMYL-PICA, 5F-CUMYL-PICA, AMB-FUBINACA, MDMB-FUBINACA, NNEI, and MN-18 during exposure to high temperatures. For. Toxicol. 37, 17–26. 10.1007/s11419-018-0430-0 30705707 PMC6349387

[B53] Kronstrand R. Guerrieri D. Vikingsson S. Wohlfarth A. Gréen H. (2018). Fatal poisonings associated with new psychoactive substances. Handb. Exp. Pharmacol. 252, 495–541. 10.1007/164_2018_110 30105471

[B54] Kumar K. K. Shalev-Benami M. Robertson M. J. Hu H. Banister S. D. Hollingsworth S. A. (2019). Structure of a signaling cannabinoid receptor 1-G protein complex. Cell 176, 448–458.e12. 10.1016/j.cell.2018.11.040. 30639101 PMC6461403

[B55] Li X. Hua T. Vemuri K. Ho J.-H. Wu Y. Wu L. (2019). Crystal structure of the human cannabinoid receptor CB1. Cell 176, 459–467. 10.1016/j.cell.2018.12.011 30639103 PMC6713262

[B56] Liu C. Jia W. Hua Z. Qian Z. (2017). Identification and analytical characterization of six synthetic cannabinoids NNL-3, 5F–NPB-22-7N, 5F–AKB-48-7N, 5F–EDMB-PINACA, EMB-FUBINACA, and EG-018. Drug Test. Anal. 9, 1251–1261. 10.1002/dta.2160. 28063270

[B57] Longworth M. Banister S. D. Boyd R. Kevin R. C. Connor M. McGregor I. S.. (2017). Pharmacology of cumyl-carboxamide synthetic cannabinoid new psychoactive substances (NPS) CUMYL-BICA, CUMYL-PICA, CUMYL-5F-PICA, CUMYL-5F-PINACA, and their analogues. ACS Chem. Nerosci. 8, 2159–2167. 10.1021/acschemneuro.7b00267 28792725

[B58] Lorenzen E. Sakmar T. P. (2019). Receptor structures for a caldron of cannabinoids. Cell 176, 409–411. 10.1016/j.cell.2019.01.012 30682366

[B59] Mardal M. Dalsgaard P. W. Qi B. Mollerup C. B. Annaert P. Linnet K. (2018). Metabolism of the synthetic cannabinoids AMB-CHMICA and 5C-AKB48 in pooled human hepatocytes and rat hepatocytes analyzed by UHPLC-(IMS)-HR-MSE. J. Chromatogr. B. 1083, 189–197. 10.1016/j.jchromb.2018.03.016 29549742

[B60] Metternich S. Zörntlein S. Schönberger T. Huhn C. (2019). Ion mobility spectrometry as a fast screening tool for synthetic cannabinoids to uncover drug trafficking in jail via herbal mixtures, paper, food, and cosmetics. Drug Test Anal. 1–14. [Epub ahead of print]. 10.1002/dta.2565 30610761

[B61] Moosmann B. Angerer V. Auwärter V. (2015). Inhomogeneities in herbal mixtures: a serious risk for consumers. For. Toxicol. 33, 54–60. 10.1007/s11419-014-0247-4

[B62] National Offender Management Service (2017). North West ‘Through the Gate Substance Misuse Services' Drug Testing Project – Further Public Health Monitoring Study – North West Final Report. Available online at: https://www.lgcgroup.com/media/1795/noms-final-phm-report-version-5.pdf (accessed February 2, 2019).

[B63] Noble C. Cannaert A. Linnet K. Stove C. (2019). Application of an activity-based receptor bioassay to investigate the in vitro activity of selected indole- and indazole-3-carboxamide-based synthetic cannabinoids at CB1 and CB2 receptors. Drug Test. Anal. 11, 501–511. 10.1002/dta.2517 30280499

[B64] Peace M. R. Krakowiak R. I. Wolf C. E. Poklis A. Poklis J. L. (2017). Identification of MDMB-FUBINACA in commercially available e-liquid formulations sold for use in electronic cigarettes. For. Sci. Int. 271:92–97. 10.1016/j.forsciint.2016.12.031 28076838 PMC5511053

[B65] Pertwee R. G. (1997). The pharmacology of CB1 and CB2 receptors. Pharmacol. Ther. 74, 129–180. 10.1016/S0163-7258(97)82001-3 9336020

[B66] Pertwee R. G. (2008). The diverse CB1 and CB2 receptor pharmacology of three plant cannabinoids: delta-9-tetrahydrocannabinol, cannabidiol and delta-9-tetrahydrocannabivarin. Br. J. Pharmacol. 153, 199–215. 10.1038/sj.bjp.0707442 17828291 PMC2219532

[B67] Pertwee R. G. Howlett A. C. Abood M. E. Alexander S. P. Di Marzo V. Elphick M. R. (2010). Cannabinoid receptors and their ligands: beyond CB1 and CB2. Pharmacol. Rev. 62, 588–631. 10.1124/pr.110.003004 21079038 PMC2993256

[B68] Pützl M. Schneiders S. Auwärter V. Münster-Müller S. Scheid N. (2015). The EU-Project ‘SPICE-Profiling' (2015-2017) - Objectives and Results of a First Study on Spice Products Containing 5F-PB-22. Toxichem Krimtech 82 (Special Issue):273. Available online at: https://www.gtfch.org/cms/images/stories/media/tb/tb2015/Puetz_et_al_2015.pdf (accessed February 9, 2019).

[B69] Ralphs R. Williams L. Askew R. Norton A. (2017). Adding spice to the porridge: the development of a synthetic cannabinoid market in an english prison. Int. J. Drug Pol. 40, 57–69. 10.1016/j.drugpo.2016.10.003 27955961

[B70] Sachdev S. Vemuri K. Banister S. D. Longworth M. Kassiou M. Santiago M. (2018). In vitro determination of the CB1 efficacy of illicit synthetic cannabinoids. *bioRxiv* 385583 [Preprint]. 10.1101/385583 PMC6965679 31412133

[B71] Schoeder C. T. Hess C. Madea B. Meiler J. Müller C. E. (2018). Pharmacological evaluation of new constituents of “Spice”: synthetic cannabinoids based on indole, indazole, benzimidazole and carbazole scaffolds. Forensic Toxicol. 36, 385–403. 10.1007/s11419-018-0415-z 29963207 PMC6002460

[B72] Sherpa D. Paudel B. M. Subedi B. H. Chow R. D. (2015). Synthetic cannabinoids: the multi-organ failure and metabolic derangements associated with getting high. J. Commun. Hosp. Intern. Med. Perspect. 5:27540. 10.3402/jchimp.v5.27540 26333853 PMC4558292

[B73] Shevyrin V. Melkozerov V. Nevero A. Eltsov O. Shafran Y. Morzherin Y.. (2015). Identification and analytical characteristics of synthetic cannabinoids with an indazole-3-carboxamide structure bearing a N-1-methoxycarbonylalkyl group. Anal. Bioanal. Chem. 407, 6301–6315. 10.1007/s00216-015-8612-7 25893797

[B74] Silva B. Fernandes C. Guedes de Pinho P. Remião F. (2018). Chiral resolution and enantioselectivity of synthetic cathinones: a brief review. J. Anal. Toxicol. 42, 17–24. 10.1093/jat/bkx074 28977427

[B75] Taschwer M. Grascher J. Schmid M. G. (2017). Development of an enantioseparation method for novel psychoactive drugs by HPLC using a Lux® Cellulose-2 column in polar organic phase mode. For. Sci. Int. 270, 232–240. 10.1016/j.forsciint.2016.10.011 28029499

[B76] Uchiyama N. Matsuda S. Wakana D. Kikura-Hanajiri R. Goda Y. (2013). New cannabimimetic indazole derivatives, N-(1-amino-3-methyl-1-oxobutan-2-yl)-1-pentyl-1H-indazole-3-carboxamide (AB-PINACA) and N-(1-amino-3-methyl-1-oxobutan-2-yl)-1-(4-fluorobenzyl)-1H-indazole-3-carboxamide (AB-FUBINACA) identified as designer drugs in illegal products. For. Toxicol. 31, 93–100. 10.1007/s11419-012-0171-4

[B77] UNODC (2015). October 2015–China: China Announces Controls Over 116 New Psychoactive Substances. Available online at: https://www.unodc.org/LSS/Announcement/Details/83b02e73-4896-4ed5-944c-51a7646647aa (accessed November 1, 2018).

[B78] UNODC (2018a). World Drug Report 2018. United Nations publication. Available online at: https://www.unodc.org/wdr2018 (accessed February 2, 2019).

[B79] UNODC (2018b). China Places Additional 32 New Psychoactive Substances Under National Control. Available online at : https://www.unodc.org/LSS/Announcement/Details/e4decfc2-0913-4a68-bbcf-24972690b698 (accessed February 2, 2019).

[B80] UNODC (2018c). November 2018–UNODC: Commission on Narcotic Drugs Decision on International Control of 4-Fluoramphetamine (4-FA), AB-PINACA, AB-CHMINACA, 5F-PB-22, UR-144 and 5F-MDMB-PINACA (5F-ADB) Enters Into Force. Available online at: https://www.unodc.org/LSS/Announcement/Details/2a0dd30f-c322-4f89-bd4d-90a0e5196314 (accessed December 6, 2019).

[B81] Weber C. Pusch S. Schollmeyer D. Münster-Müller S. Pütz M. Opatz T. (2016). Characterization of the synthetic cannabinoid MDMB-CHMCZCA. Beils. J. Org. Chem. 12, 2808–2815. 10.3762/bjoc.12.279 28144353 PMC5238538

[B82] Wilson C. D. Tai S. Ewing L. Crane J. Lockhart T. Fujiwara R. (2019). CB1R-mediated convulsant effects of synthetic cannabinoids. J. Pharmacol. Expt Ther. 368,146–156. 10.1124/jpet.118.251157 PMC6323622 30420360

[B83] Xue Y.-P. Cao C.-H. Zheng Y. G. (2018). Enzymatic asymmetric synthesis of chiral amino acids. Chem. Soc. Rev. 47, 1516–1561. 10.1039/C7CS00253J 29362736

