# Supplementary material for: Enantiospecific Synthesis, Chiral Separation, and Biological Activity of Four Indazole-3-Carboxamide-Type Synthetic Cannabinoid Receptor Agonists and Their Detection in Seized Drug Samples
Source: Front Chem. 2019 May 16;7:321. doi: 10.3389/fchem.2019.00321 (PMC6532652; doi:10.3389/fchem.2019.00321)
Supplement: Supplementary file 1 [file Data_Sheet_1.docx]

**Enantiospecific synthesis, chiral separation and biological activity of four indazole-3-carboxamide-type synthetic cannabinoid receptor agonists and their detection in seized drug samples**

Lysbeth H Antonides^1,2^, Annelies Cannaert^3,4^, Caitlyn Norman^2^, Loelia Vives^2,5^, Aidan Harrison^6^, Andrew Costello^7,8^, Niamh Nic Daeid^1,2^, Christophe P. Stove^3*^, Oliver B. Sutcliffe^7,9*^, Craig McKenzie^2*^

^1^ Leverhulme Research Centre for Forensic Science, University of Dundee, United Kingdom; ^2^ Forensic Drug Research Group, Centre for Anatomy and Human Identification, University of Dundee, Dundee, United Kingdom; ^3^ Laboratory of Toxicology, Faculty of Pharmaceutical Sciences, Ghent University, Ghent, Belgium; ^4^ Laboratory of Toxicology, National Institute of Criminalistics and Criminology, Brussels, Belgium; ^5^ IUT "A" Paul Sabatier, Département de Chimie, Castres, France; ^6^ Phenomenex, Macclesfield, United Kingdom; ^7^ Manchester Drug Analysis and Knowledge Exchange, Manchester Metropolitan University, Manchester, United Kingdom; ^8^ Greater Manchester Police, Openshaw Complex, Lawton Street, Openshaw, Manchester, United Kingdom; ^9^ Division of Chemistry and Environmental Science, Manchester Metropolitan University, Manchester, United Kingdom.

**Supplementary Information**

**Reference standard characterisation data**

(*R*)-AB-FUBINACA: ^1^H-NMR (400 MHz, CDCl_3_) δ (ppm) 8.32 (d, J = 8.2 Hz, 1H), 7.51 (d, J = 9.2 Hz, 1H), 7.35-7.38 (m, 1H), 7.26-7.32 (m, 2H), 7.18 (dd, J = 8.7, 5.5 Hz, 2H), 6.99 (t, J = 8.7 Hz, 2H), 6.26 (s, 1H, NH_2_), 5.57 (s, 1H, -NH_2_), 5.57 (s, 2H), 4.54 (dd, J = 8.7, 6.9 Hz, 1H), 2.35 (q, J = 6.9 Hz, 1H), 1.07 (q, J = 3.5 Hz, 6H)**.** ^13^C-NMR (101 MHz, CDCl_3_) δ (ppm) 173.50 (s, 1C), 163.78 - 161.33 (d, J_CF_ = 247.5. Hz, 1C), 162.86 (s, 1C), 140.86 (s, 1C), 137.15 (s, 1C), 131.68 (d, J = 2.9 Hz, 1C), 129.13 (s, 1C), 129.04 (s, 1C), 127.24 (s, 1C), 123.31 (s, 1C), 123.09 (s, 1C), 122.79 (s, 1C), 116.03 (s, 1C), 115.82 (s, 1C), 109.64 (s, 1C), 57.91 (s, 1C), 53.04 (s, 1C), 30.67 (s, 1C), 19.59 (s, 1C), 18.32 (s, 1C). GC-MS (EI) principal ions - mass (% of base peak): 109 (100), 253 (62), 324 (51), 254 (12) 325 (11). M^+^ at *m/z* = 368; HRMS (ESI+) 368.1650. UV (302 nm). See Figure S1 for MS/MS and UV spectra.

(*S*)-AB-FUBINACA: ^1^H-NMR (400 MHz, CDCl_3_) δ (ppm) 8.31 (d, J = 8.2 Hz, 1H), 7.52 (d, J = 8.7 Hz, 1H), 7.36 (dd, J = 8.0, 7.1 Hz, 1H), 7.29 (q, J = 7.9 Hz, 2H), 7.18 (dd, J = 8.5, 5.3 Hz, 2H), 6.99 (t, J = 8.7 Hz, 2H), 6.34 (s, 1H, -NH_2_), 5.63 (s, 1H, -NH_2_), 5.56 (s, 2H), 4.55 (dd, J = 9.2, 6.9 Hz, 1H), 2.34 (q, J = 6.9 Hz, 1H), 1.07 (q, J = 3.5 Hz, 6H). ^13^C-NMR (101 MHz, CDCl_3_) δ (ppm)173.55 (s, 1C), 163.73 – 161.31 (d, J_CF_ = 244.4 Hz, 1C), 162.85 (s, 1C), 140.86 (s, 1C), 137.16 (s, 1C), 131.66 (s, 1C), 129.13 (s, 1C), 129.04 (s, 1C), 127.24 (s, 1C), 123.31 (s, 1C), 123.08 (s, 1C), 122.78 (s, 1C), 116.03 (s, 1C), 115.81 (s, 1C), 109.64 (s, 1C), 57.91 (s, 1C), 53.03 (s, 1C), 30.71 (s, 1C), 19.58 (s, 1C), 18.32 (s, 1C). GC-MS (EI) principal ions - mass (% of base peak): 109 (100), 253 (58), 324 (47), 254 (11) 325 (11); HRMS (ESI+) 368.1652; UV (301 nm). See Figure S1 for MS/MS and UV spectra.

(*R*)-AB-CHMINACA: ^1^H-NMR (400 MHz, CDCl_3_) δ (ppm)8.28 (d, J = 8 Hz, 1H), 7.50 (d, J = 8.7 Hz, 1H), 7.39 (ddd, J = 13.5, 8.0, 0.9 Hz, 2H), 7.23-7.26 (m, 1H), 6.50 (br. s, 1H), 5.72 (br. s, 1H), 4.56 (dd, J = 9.2, 6.9 Hz, 1H), 4.19 (d, J = 7.3 Hz, 2H), 2.34 (q, J = 6.7 Hz, 1H), 1.97-2.03 (m, 1H), 1.67 (d, J = 8.7 Hz, 3H), 1.58 (t, J = 14.9 Hz, 2H), 1.13-1.24 (m, 3H), 1.07 (q, J = 3.4 Hz, 6H), 1.01 (dd, J = 8.5, 3.4 Hz, 2H)**.** ^13^C-NMR (101 MHz, CDCl_3_) δ (ppm) 173.62 (s, 1C), 163.14 (s, 1C), 141.48 (s, 1C), 136.39 (s, 1C), 126.74 (s, 1C), 123.01-122.74 (1C), 122.73-122.64 (1C), 122.55 (s, 1C), 109.70 (s, 1C), 57.93 (s, 1C), 55.85 (s, 1C), 38.80 (s, 1C), 31.01 (s, 1C), 30.99-30.87 (1C), 30.46 (s, 1C), 26.28 (s, 1C), 25.72 (s, 1C), 25.70-25.61 (1C), 19.61 (s, 1C), 18.28 (s, 1C); GC-MS (EI) principal ions - mass (% of base peak): 241 (100), 312 (84), 145 (58), 55 (28) 313 (18); HRMS (ESI+) 356.221; UV (304 nm). See Figure S2 for MS/MS and UV spectra.

(*S*)-AB-CHMINACA: ^1^H-NMR (400 MHz, CDCl_3_) δ (ppm) 8.29-8.31 (m, 1H), 7.48 (d, J = 8.7 Hz, 1H), 7.37-7.42 (m, 2H), 7.23-7.27 (m, 1H), 6.29 (s, 1H), 5.53 (s, 1H), 4.51 (dd, J = 8.7, 6.9 Hz, 1H), 4.20 (d, J = 7.3 Hz, 2H), 2.36 (q, J = 6.7 Hz, 1H), 1.98-2.03 (m, 1H), 1.69 (s, 3H), 1.59 (t, J = 15.3 Hz, 2H), 1.14-1.24 (m, 3H), 1.07 (dd, J = 6.9, 2.3 Hz, 6H), 1.00-1.03 (m, 2H) ^13^C-NMR (101 MHz, CDCl_3_) δ (ppm) 173.79 (s, 1C), 163.12 (s, 1C), 141.48 (s, 1C), 136.40 (s, 1C), 126.72 (s, 1C), 122.74 (s, 1C), 122.71 (s, 1C), 122.51 (s, 1C), 109.71 (s, 1C), 57.90 (s, 1C), 55.84 (s, 1C), 38.80 (s, 1C), 31.00 (s, 1C), 30.97 (s, 1C), 30.65 (s, 1C), 26.28 (s, 1C), 25.72 (s, 1C), 25.68 (s, 1C), 19.59 (s, 1C), 18.33 (s, 1C). GC-MS (EI) principal ions - mass (% of base peak): 241 (100), 312 (83), 145 (59), 55 (28) 313 (18); HRMS (ESI+) 356.2214; UV (304 nm). See Figure S2 for MS/MS and UV spectra.

(*R*)-5F-MDMB-PINACA***:*** ^1^H-NMR (400 MHz, CDCl_3_) δ (ppm) 8.33 (dd, J = 8.2, 0.9 Hz, 1H), 7.47-7.62 (m, 1H), 7.37-7.44 (m, 2H), 7.22-7.29 (m, 1H), 4.68-4.78 (m, 1H), 4.46-4.51 (t, 1H), 4.41 (t, J = 7.1 Hz, 2H), 4.34-4.38 (t, 1H), 3.69-3.80 (s, 3H), 1.96-2.04 (m, 2H), 1.67-1.81 (m, 2H), 1.41-1.50 (m, 2H), 1.01-1.12 (s, 9H). ^13^C-NMR (101 MHz, CDCl_3_) δ (ppm) 172.29 (s, 1C), 162.40 (s, 1C), 140.88 (s, 1C), 136.79 (s, 1C), 126.82 (s, 1C), 122.98 (s, 1C), 122.91 (s, 1C), 122.72 (s, 1C), 109.22 (s, 1C), 83.84 (d, J_CF_ = 164.9 Hz, 1C), 59.54 (s, 1C), 51.93 (s, 1C), 49.27 (s, 1C), 35.15 (s, 1C), 29.88 (s, 1C), 29.43 (s, 1C), 26.76 (s, 3C), 22.76 (d, J_CF_ = 4.8 Hz, 1C). GC-MS (EI) principal ions - mass (% of base peak): 233 (100), 145 (55), 131 (52), 289 (42) 321 (38); HRMS (ESI+) 377.2111; UV (304 nm). See Figure S3 for MS/MS and UV spectra.

(*S*)-5F-MDMB-PINACA: ^1^H-NMR (400 MHz, CDCl_3_) δ (ppm) 8.33 (d, J = 8.2 Hz, 1H), 7.54 (d, J = 10.1 Hz, 1H), 7.40-7.41 (m, 2H), 7.23-7.27 (m, 1H), 4.72 (d, J = 9.6 Hz, 1H), 4.48 (t, J = 6.0 Hz, 1H), 4.41 (t, J = 7.1 Hz, 2H), 4.37 (t, J = 6.0 Hz, 1H), 3.75 (s, 3H), 2.00 (t, J = 7.6 Hz, 2H), 1.72 (d, J = 25.6 Hz, 2H), 1.46 (t, J = 7.8 Hz, 2H), 1.08 (s, 9H)**.** ^13^C-NMR (101 MHz, CDCl_3_) δ (ppm) 172.29 (s, 1C), 162.39 (s, 1C), 140.88 (s, 1C), 136.80 (s, 1C), 126.83 (s, 1C), 122.98 (s, 1C), 122.91 (s, 1C), 122.72 (s, 1C), 109.22 (s, 1C), 83.84 (d, J_CF_ = 164.9 Hz, 1C), 59.54 (s, 1C), 51.93 (s, 1C), 49.27 (s, 1C), 35.14 (s, 1C), 29.98 (d, J_CF_ = 20.1 Hz, 1C), 29.42 (s, 1C), 26.76 (s, 3C), 22.76 (d, J = 4.8 Hz, 1C). GC-MS (EI) principal ions - mass (% of base peak): 233(100), 145 (63), 131 (55), 289 (41) 321 (41); HRMS (ESI+) 377.2115; UV (304 nm). See Figure S3 for MS/MS and UV spectra.

(*R*)-AMB-FUBINACA: ^1^H-NMR (400 MHz, DMSO-d6) δ (ppm) 8.20 (d, J = 8.2 Hz, 1H), 8.10 (d, J = 8.2 Hz, 1H), 7.76 (d, J = 8.2 Hz, 1H), 7.42 (t, J = 7.3 Hz, 1H), 7.23-7.32 (m, 3H), 7.11-7.15 (m, 2H), 5.74 (s, 2H), 4.38 (dd, J = 8.0, 7.1 Hz, 1H), 3.64 (s, 3H), 2.22 (q, J = 6.7 Hz, 1H), 0.89-0.93 (m, 6H) ^13^C-NMR (101 MHz, DMSO-D6) δ (ppm) 172.56 (s, 1C), 163.38 - 160.95 (d, J_CF_ 245.43 Hz, 1 C), 162.41 (s, 1C), 141.03 (s, 1C), 137.40 (s, 1C), 133.55 (s, 1C), 130.05 (s, 1C), 129.97 (s, 1C), 127.56 (s, 1C), 123.31 (s, 1C), 122.94 (s, 1C), 122.25 (s, 1C), 116.16 (s, 1C), 115.95 (s, 1C), 111.14 (s, 1C), 57.95 (s, 1C), 52.39 (s, 1C), 52.14 (s, 1C), 30.31 (s, 1C), 19.64 (s, 1C), 19.34 (s, 1C). GC-MS (EI) principal ions - mass (% of base peak): 109 (100), 253 (62), 324 (27), 254 (11), 269 (9.3). M^+^ at m/z 383; HRMS (ESI+) 383.1644; UV (302 nm). See Figure S4 for MS/MS and UV spectra.

(*S*)-AMB-FUBINACA: ^1^H-NMR (400 MHz, CDCl_3_) δ (ppm) 8.36 (d, J = 8.2 Hz, 1H), 7.48 (d, J = 8.7 Hz, 1H), 7.35-39 (m, 1H), 7.25-7.32 (m, 2H), 7.18 (dd, J = 8.2, 5.5 Hz, 2H), 6.99 (t, J = 8.5 Hz, 2H), 5.58 (s, 2H), 4.80 (dd, J = 8.9, 5.3 Hz, 1H), 3.77 (s, 3H), 2.30 (q, J = 6.4 Hz, 1H), 1.03 (t, J = 6.6 Hz, 6H). (101MHz, CDCl_3_) δ (ppm) 172.70 (s, 1C), 163.75 – 161.30 (d, J_CF_ = 247.45 Hz, 1C) 162.47 (s, 1C), 140.84 (s, 1C), 137.36 (s, 1C), 131.78 (s, 1C), 129.07 (s, 1C), 128.99 (s, 1C), 127.17 (s, 1C), 123.38 (s, 1C), 123.00 (s, 2C), 116.00 (s, 1C), 115.79 (s, 1C), 109.53 (s, 1C), 56.85 (s, 1C), 53.00 (s, 1C), 52.31 (s, 1C), 31.68 (s, 1C), 19.25 (s, 1C), 18.17 (s, 1C).GC-MS (EI) principal ions - mass (% of base peak): 109 (100), 253 (59), 324 (27), 254 (9.9), 269 (9.0). M^+^ at m/z 383; HRMS (ESI+) 383.1649; UV (302 nm). See Figure S4 for MS/MS and UV spectra.

**Figure S1** HPLC-PDA-QToF-MS/MS characterisation data for (*R*)- and (*S*)-AB-FUBINACA **(1)**


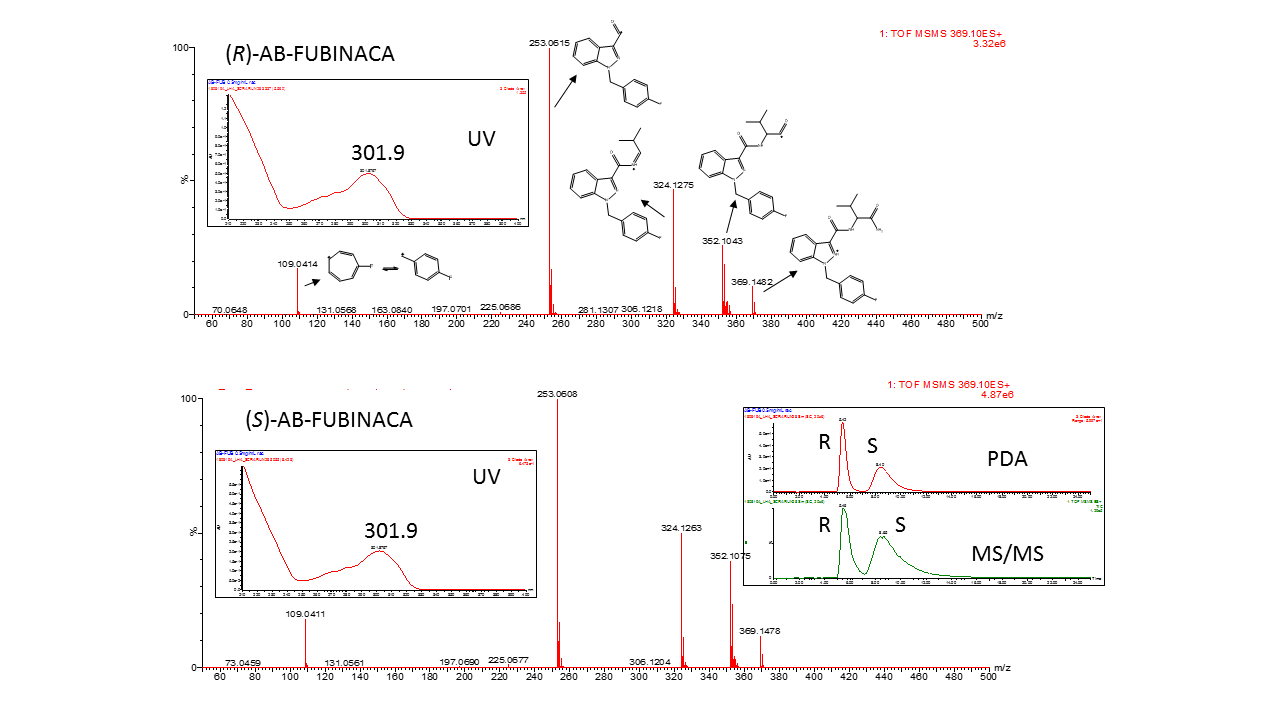


**Figure S2** HPLC-PDA-QToF-MS/MS characterisation data for (*R*)- and (*S*)-AB-CHMINACA **(4)**


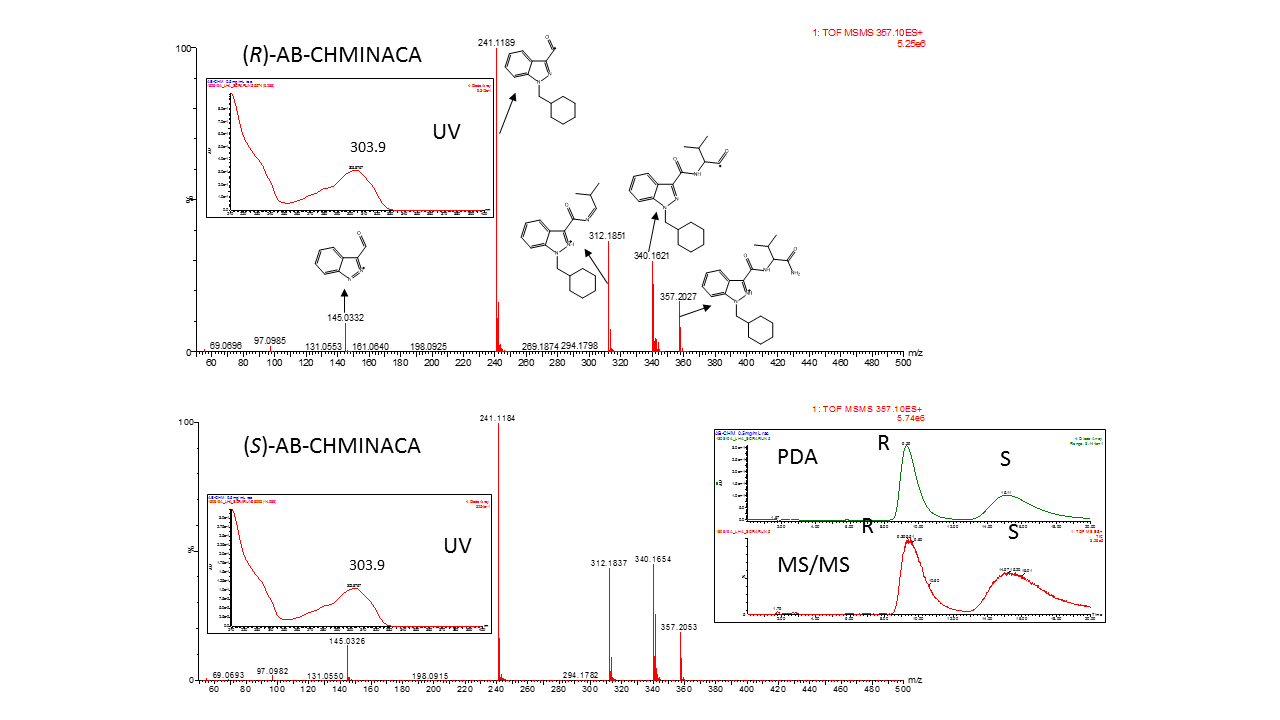


**Figure S3** HPLC-PDA-QToF-MS/MS characterisation data for (*R*)- and (*S*)-5F-MDMB PINACA **(3)**


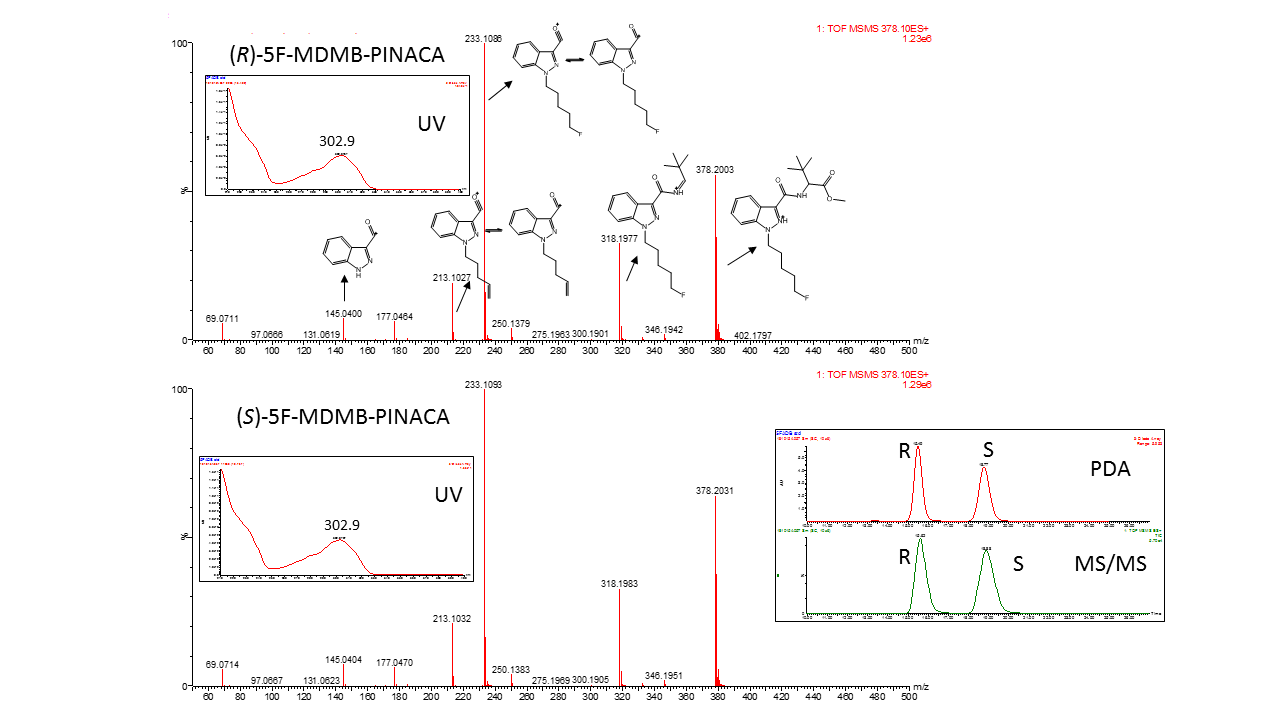


**Figure S4** HPLC-PDA-QToF-MS/MS characterisation data for (*R*)- and (*S*)-AMB-FUBINACA **(2)**


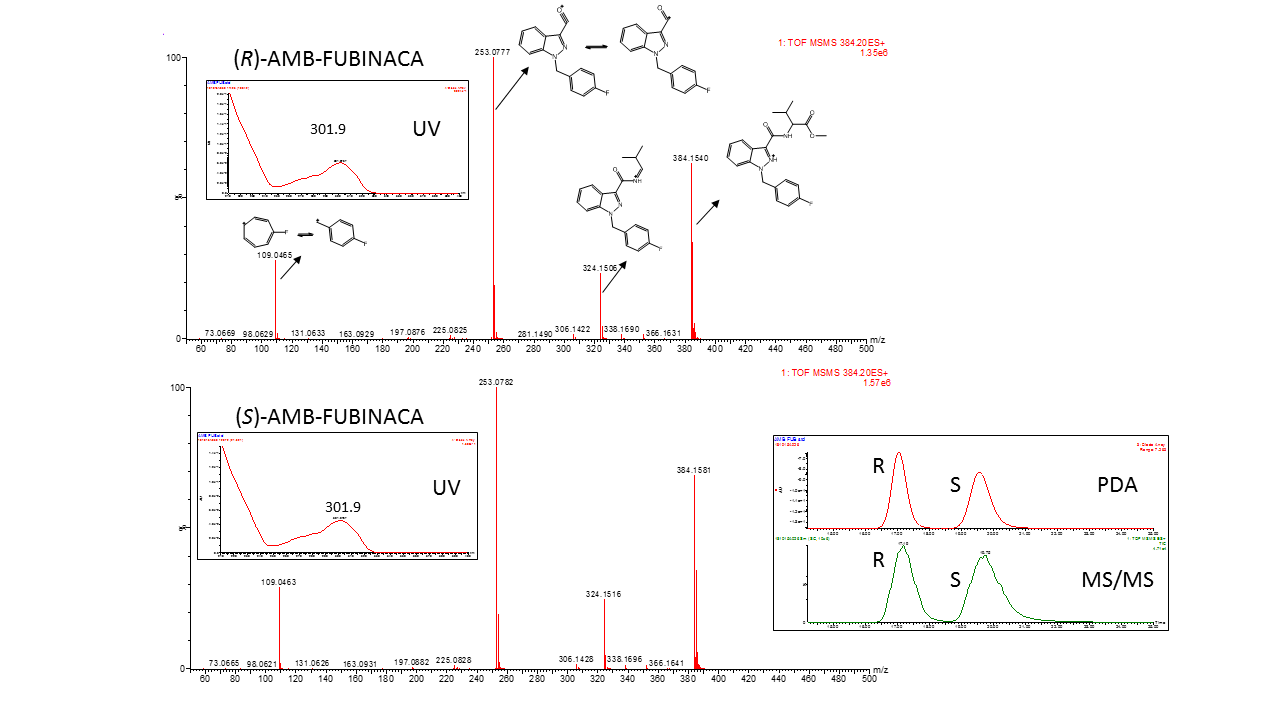


**Table S1**

Optimisation of Phenomenex Lux Chiral HPLC columns for indazole-3-carboxamide synthetic cannabinoid receptor agonist (SCRA) enantiomer separation (n=3)

|  | HPLC Method  H_2_O:ACN | **Lux Amylose-1** | | | | | | **i-Cellulose-5** | | | | | |
| --- | --- | --- | --- | --- | --- | --- | --- | --- | --- | --- | --- | --- | --- |
|  |  | RT (mins) | Peak Symmetry | N | k | α | Rs | RT (mins) | Peak  Symmetry | N | k | α | Rs |
| AB-FUBINACA **(1)** | 50:50 | 5.474 | 0.719 | 1205.1 | 1.81 | 1.83 | 2.54 | 6.947 | 0.772 | 6426.3 | 2.61 | 1.45 | 4.25 |
|  |  | 8.402 | 0.610 | 346.2 | 3.31 |  |  | 9.231 | 0.640 | 4717.8 | 3.79 |  |  |
|  | 55:45 | 7.587 | 0.674 | 1118.8 | 2.90 | 1.85 | 2.85 | 9.900 | 0.757 | 6471.9 | 4.09 | 1.47 | 4.59 |
|  |  | 12.37 | 0.671 | 500.1 | 5.35 |  |  | 13.61 | 0.597 | 4536.8 | 6.00 |  |  |
|  | 45:55 | 4.330 | 0.644 | 1132.9 | 1.18 | 1.85 | 2.09 | 5.344 | 0.758 | 6003.3 | 1.82 | 1.45 | 3.74 |
|  |  | 6.313 | 0.582 | 296.9 | 2.18 |  |  | 6.887 | 0.637 | 4463.0 | 2.64 |  |  |
| AB-CHMINACA **(4)** | 50:50 | 9.235 | 0.544 | 788.4 | 3.75 | 1.77 | 2.42 | 12.70 | 0.733 | 6319.3 | 5.58 | 1.48 | 4.66 |
|  |  | 14.87 | 0.598 | 453.1 | 6.64 |  |  | 17.91 | 0.501 | 4103.8 | 8.28 |  |  |
|  | 55:45 | 13.84 | 0.517 | 654.4 | 6.12 | 1.78 | 2.33 | 19.46 | 0.701 | 6329.9 | 9.01 | 1.49 | 4.82 |
|  |  | 23.11 | 0.519 | 852.6 | 10.9 |  |  | 28.02 | 0.444 | 3946.8 | 13.4 |  |  |
|  | 45:55 | 6.788 | 0.560 | 737.8 | 2.44 | 1.78 | 2.10 | 9.133 | 0.727 | 5895.7 | 3.83 | 1.48 | 4.25 |
|  |  | 10.52 | 0.579 | 353.0 | 4.33 |  |  | 12.60 | 0.496 | 3832.3 | 5.66 |  |  |
| 5F-MDMB-PINACA **(3)** | 50:50 | 11.31 | 0.767 | 3146.9 | 4.80 | 1.27 | 2.44 | 15.85 | 0.839 | 10538.7 | 7.21 | 1.09 | 1.93 |
|  |  | 13.80 | 0.740 | 2074.1 | 6.08 |  |  | 17.14 | 0.846 | 10400.7 | 7.89 |  |  |
|  | 55:45 | 19.28 | 0.758 | 4050.6 | 8.91 | 1.27 | 3.06 | 27.83 | 0.818 | 10797.7 | 13.3 | 1.10 | 2.20 |
|  |  | 24.00 | 0.739 | 3327.6 | 11.33 |  |  | 30.35 | 0.808 | 11179.2 | 14.6 |  |  |
|  | 45:55 | 7.481 | 0.742 | 2724.5 | 2.79 | 1.26 | 2.00 | 10.30 | 0.834 | 9305.1 | 4.44 | 1.10 | 1.74 |
|  |  | 8.932 | 0.732 | 1691.9 | 3.53 |  |  | 11.11 | 0.826 | 9065.3 | 4.86 |  |  |
| AMB-FUBINACA **(2)** | 50:50 | 13.81 | 0.737 | 2858.1 | 6.09 | 1.16 | 1.60 | 17.72 | 0.871 | 10485.4 | 8.20 | 1.05 | 1.00 |
|  |  | 15.73 | 0.674 | 1755.1 | 7.08 |  |  | 18.49 | 0.816 | 9363.0 | 8.60 |  |  |
|  | 55:45 | 23.92 | 0.731 | 3678.0 | 11.29 | 1.17 | 1.99 | 31.24 | 0.837 | 11064.7 | 15.1 | 1.05 | 1.15 |
|  |  | 27.57 | 0.650 | 3012.8 | 13.16 |  |  | 32.73 | 0.800 | 10147.0 | 15.8 |  |  |
|  | 45:55 | 8.971 | 0.758 | 2508.9 | 3.51 | 1.16 | 1.34 | 11.21 | 0.899 | 10147.8 | 4.94 | 1.05 | 0.86 |
|  |  | 10.08 | 0.674 | 1414.6 | 4.07 |  |  | 11.65 | 0.790 | 8632.3 | 5.17 |  |  |

N- column efficiency; k – capacity factor, α – selectivity factor; Rs – peak resolution, numbers in parenthesis refer to Compound numbering provided in Figure 1.

**Calculation of Intrinsic Potency value**

When the relative proportions of the enantiomers have not been determined:

$$Estimated Intrinsic Potency= \frac{\left[ \left( \frac{{CB1 EC}_{50, JWH-018}}{CB1 {EC}_{50, SCRA}} \right)x {Conc}_{SCRA} \right]}{100}$$

*Eqn. 1*

Where,

EC_50_ values are expressed in nM, Conc_SCRA_ is the concentration (mg/g) of a particular SCRA detected in the sampe.

Where multiple SCRAs are present in a single sample the term on the top line is simply repeated for each SCRA detected and added together.

When the relative proportions of the enantiomers have been determined they can be used to adjust the intrinsic potency value:

$$Estimated Intrinsic Potency= \frac{\left[ \left( \frac{{CB1 EC}_{50, JWH-018}}{CB1 {EC}_{50, SCRA}} \right)x P(S) \right]+\left[ \left( \frac{{CB1 EC}_{50, JWH-018}}{{CB1 EC}_{50, SCRA}} \right)x P(R) \right]*{Conc}_{SCRA}}{100}$$

*Eqn. 2*

Where,

EC_50_ values are expressed in nM, Conc_SCRA_ is the concentration (mg/g) of a particular SCRA detected in the sample, P(*S*) is the proportion of the (*S*)-enantiomer present in the sample e.g. 93.6% = 0.936, P(*R*) is the proportion of the (*R*)-enantiomer present in the sample e.g. 6.4% = 0.064.

**Table S2** Calculation of Relative Potency values used in this study:

| Analyte | EC50 (nM) or other factor | Relative Potency Factor | Reference |
| --- | --- | --- | --- |
| JWH-018 | 45.1 | 1 | This study |
| 5F-MDMB-PINACA | 1.78 | 25.34 |  |
| AMB-FUBINACA | 9.11 | 4.95 |  |
| JWH-018 | 23.9 | 1 | Cannaert et al., 2017 |
| ADB-CHMINACA | 1.49 | 16.04 |  |
| AMB-CHMICA | 25.34 x (0.59/3.5)* | n.a ^$^ | Banister et al., 2016 |

* (EC_50, 5F-MDMB-PINACA_ / EC_50 AMB-CHMICA_) as reported by Banister et al. (2016).

^$^ unavailable as JWH-018 was not used as a positive control by Banister et al. (2016) who instead use CP 55,490 in the cited study.

**Table S3a** Calculation of Intrinsic Potency values for seized samples reported in this study

| Sample ID | Replicate | SCRA1 detected | Enantiomer Ratio  S:R | Relative Potency Factor  100% (*S*) | Relative Potency Factor  100% (*R*) | Corrected (*S*)  Potency Factor | Corrected (*R*)  Potency Factor | Corrected Total  Potency Factor  (SCRA1) | SCRA2 (Achiral) | Relative Potency Factor  100%  (*S*) | Concentration SCRA1  (mg/g) | Concentration SCRA 2  (mg/g) | Intrinsic potency |
| --- | --- | --- | --- | --- | --- | --- | --- | --- | --- | --- | --- | --- | --- |
| 1 | 1 | 5F-MDMB-PINACA | 0.936:0.064 | 25.34 | 0.3443 | 23.716 | 0.0220 | 23.74 | AMB-CHMICA | 4.164 | 16.3 | 136 | 9.52 |
| 1 | 2 | 5F-MDMB-PINACA |  | 25.34 | 0.3443 | 23.716 | 0.0220 | 23.74 | AMB-CHMICA | 4.164 | 17.2 | 145 | 10.1 |
| 1 | 3 | 5F-MDMB-PINACA |  | 25.34 | 0.3443 | 23.716 | 0.0220 | 23.74 | AMB-CHMICA | 4.164 | 15.2 | 132 | 9.10 |
| 3 | 1 | 5F-MDMB-PINACA | 0.993:0.007 | 25.34 | 0.3443 | 25.160 | 0.0024 | 25.16 | - | - | 20.0 | - | 5.03 |
| 3 | 2 | 5F-MDMB-PINACA |  | 25.34 | 0.3443 | 25.160 | 0.0024 | 25.16 | - | - | 66.1 | - | 16.6 |
| 3 | 3 | 5F-MDMB-PINACA |  | 25.34 | 0.3443 | 25.160 | 0.0024 | 25.16 | - | - | 40.2 | - | 10.1 |
| 4 | 1 | 5F-MDMB-PINACA | 0.993:0.007 | 25.34 | 0.3443 | 25.160 | 0.0024 | 25.16 | - | - | 23.0 | - | 5.78 |
| 4 | 2 | 5F-MDMB-PINACA |  | 25.34 | 0.3443 | 25.160 | 0.0024 | 25.16 | - | - | 27.4 | - | 6.89 |
| 4 | 3 | 5F-MDMB-PINACA |  | 25.34 | 0.3443 | 25.160 | 0.0024 | 25.16 | - | - | 29.4 | - | 7.39 |
| 5 | 1 | 5F-MDMB-PINACA | 0.993:0.007 | 25.34 | 0.3443 | 25.160 | 0.0024 | 25.16 | - | - | 58.8 | - | 14.8 |
| 5 | 2 | 5F-MDMB-PINACA |  | 25.34 | 0.3443 | 25.160 | 0.0024 | 25.16 | - | - | 40.1 | - | 10.1 |
| 5 | 3 | 5F-MDMB-PINACA |  | 25.34 | 0.3443 | 25.160 | 0.0024 | 25.16 | - | - | 91.5 | - | 23.0 |
| 6 | 1 | ADB-CHMINACA | 0.993:0.007 | 16.04 | - | - | - | 16.04 | - | - | 12.9 | - | 2.06 |
| 6 | 2 | ADB-CHMINACA |  | 16.04 | - | - | - | 16.04 | - | - | 14.7 | - | 2.35 |
| 6 | 3 | ADB-CHMINACA |  | 16.04 | - | - | - | 16.04 | - | - | 15.8 | - | 2.53 |
| 7 | 1 | 5F-MDMB-PINACA | 0.993:0.007 | 25.34 | 0.3443 | 25.160 | 0.0024 | 25.16 | - | - | 12.3 | - | 3.09 |
| 7 | 2 | 5F-MDMB-PINACA |  | 25.34 | 0.3443 | 25.160 | 0.0024 | 25.16 | - | - | 12.8 | - | 3.23 |
| 7 | 3 | 5F-MDMB-PINACA |  | 25.34 | 0.3443 | 25.160 | 0.0024 | 25.16 | - | - | 24.7 | - | 6.21 |
| 8 | 1 | 5F-MDMB-PINACA | 0.993:0.007 | 25.34 | 0.3443 | 25.160 | 0.0024 | 25.16 | - | - | 37.4 | - | 9.41 |
| 8 | 2 | 5F-MDMB-PINACA |  | 25.34 | 0.3443 | 25.160 | 0.0024 | 25.16 | - | - | 33.6 | - | 8.45 |
| 8 | 3 | 5F-MDMB-PINACA |  | 25.34 | 0.3443 | 25.160 | 0.0024 | 25.16 | - | - | 40.4 | - | 10.2 |
| 9 | 1 | 5F-MDMB-PINACA | 0.993:0.007 | 25.34 | 0.3443 | 25.160 | 0.0024 | 25.16 | - | - | 11.3 | - | 2.85 |
| 9 | 2 | 5F-MDMB-PINACA |  | 25.34 | 0.3443 | 25.160 | 0.0024 | 25.16 | - | - | 11.0 | - | 2.78 |
| 9 | 3 | 5F-MDMB-PINACA |  | 25.34 | 0.3443 | 25.160 | 0.0024 | 25.16 | - | - | 8.6 | - | 2.16 |

**Table S3b** Calculation of Intrinsic Potency values for seized samples reported in this study

| Sample ID | Replicate | SCRA1 detected | Enantiomer Ratio  S:R | Relative Potency Factor  100% (*S*) | Relative Potency Factor  100% (*R*) | Corrected (*S*)  Potency Factor | Corrected (*R*)  Potency Factor | Corrected Total  Potency Factor  (SCRA1) | SCRA2 (Achiral) | Relative Potency Factor  100%  (*S*) | Concentration SCRA1  (mg/g) | Concentration SCRA 2  (mg/g) | Intrinsic potency |
| --- | --- | --- | --- | --- | --- | --- | --- | --- | --- | --- | --- | --- | --- |
| 10 | 1 | AMB-FUBINACA | 0.968:0.032 | 4.95 | 0.3443 | 4.792 | 0.0259 | 4.82 | EMB-FUBINACA | 54.8 | 19.81 | - | - |
| 10 | 2 | AMB-FUBINACA |  | 4.95 | 0.3443 | 4.792 | 0.0259 | 4.82 | EMB-FUBINACA | 54.7 | 19.18 | - | - |
| 10 | 3 | AMB-FUBINACA |  | 4.95 | 0.3443 | 4.792 | 0.0259 | 4.82 | EMB-FUBINACA | 47.5 | 15.55 | - | - |
| 11 | 1 | 5F-MDMB-PINACA | 0.991:0.009 | 25.34 | 0.3443 | 25.109 | 0.0031 | 25.11 | - | 29.5 | - | 741 | 7.41 |
| 11 | 2 | 5F-MDMB-PINACA |  | 25.34 | 0.3443 | 25.109 | 0.0031 | 25.11 | - | 15.7 | - | 395 | 3.95 |
| 11 | 3 | 5F-MDMB-PINACA |  | 25.34 | 0.3443 | 25.109 | 0.0031 | 25.11 | - | 15.5 | - | 389 | 3.89 |
| 12 | 1 | AMB-FUBINACA | 0.982:0.018 | 4.95 | 0.8082 | 4.861 | 0.0145 | 4.88 | - | 45.7 | - | 223 | 2.23 |
| 12 | 2 | AMB-FUBINACA |  | 4.95 | 0.8082 | 4.861 | 0.0145 | 4.88 | - | 58.5 | - | 285 | 2.85 |
| 12 | 3 | AMB-FUBINACA |  | 4.95 | 0.8082 | 4.861 | 0.0145 | 4.88 | - | 42.8 | - | 209 | 2.09 |
| 13 | 1 | AMB-FUBINACA | 0.982:0.018 | 4.95 | 0.8082 | 4.861 | 0.0145 | 4.88 | - | 43.2 | - | 211 | 2.11 |
| 13 | 2 | AMB-FUBINACA |  | 4.95 | 0.8082 | 4.861 | 0.0145 | 4.88 | - | 45.9 | - | 224 | 2.24 |
| 13 | 3 | AMB-FUBINACA |  | 4.95 | 0.8082 | 4.861 | 0.0145 | 4.88 | - | 43.9 | - | 214 | 2.14 |
| 14 | 1 | AMB-FUBINACA | 0.982:0.018 | 4.95 | 0.8082 | 4.861 | 0.0145 | 4.88 | - | 45.4 | - | 221 | 2.21 |
| 14 | 2 | AMB-FUBINACA |  | 4.95 | 0.8082 | 4.861 | 0.0145 | 4.88 | - | 26.0 | - | 127 | 1.27 |
| 14 | 3 | AMB-FUBINACA |  | 4.95 | 0.8082 | 4.861 | 0.0145 | 4.88 | - | 32.3 | - | 158 | 1.58 |
| 15 | 1 | AMB-FUBINACA | 0.982:0.018 | 4.95 | 0.8082 | 4.861 | 0.0145 | 4.88 | - | 39.0 | - | 190 | 1.90 |
| 15 | 2 | AMB-FUBINACA |  | 4.95 | 0.8082 | 4.861 | 0.0145 | 4.88 | - | 40.2 | - | 196 | 1.96 |
| 15 | 3 | AMB-FUBINACA |  | 4.95 | 0.8082 | 4.861 | 0.0145 | 4.88 | - | 40.4 | - | 197 | 1.97 |
| 16 | 1 | AMB-FUBINACA | 0.982:0.018 | 4.95 | 0.8082 | 4.861 | 0.0145 | 4.88 | - | 42.6 | - | 208 | 2.08 |
| 16 | 2 | AMB-FUBINACA |  | 4.95 | 0.8082 | 4.861 | 0.0145 | 4.88 | - | 34.7 | - | 169 | 1.69 |
| 16 | 3 | AMB-FUBINACA |  | 4.95 | 0.8082 | 4.861 | 0.0145 | 4.88 | - | 33.9 | - | 165 | 1.65 |
